# Manufacturing and Properties of Jute Fiber-Reinforced Polymer Composites—A Comprehensive Review

**DOI:** 10.3390/ma18051016

**Published:** 2025-02-25

**Authors:** Raiyan Mohammad Iqbal, Raju Ahammad, Md Arifuzzaman, Md Shariful Islam, Md Mainul Islam

**Affiliations:** 1 Department of Mechanical Engineering, Khulna University of Engineering & Technology, Khulna 9203, Bangladesh; raiyanmohammadiqbal@gmail.com (R.M.I.); rajuahammad@me.kuet.ac.bd (R.A.); arif48@me.kuet.ac.bd (M.A.); msislam@me.kuet.ac.bd (M.S.I.); 2 School of Engineering and Centre for Future Materials, University of Southern Queensland, Toowoomba, QLD 4350, Australia

**Keywords:** jute fiber-reinforced composites, jute fiber, polymer matrix, manufacturing process, mechanical properties

## Abstract

Jute fiber-reinforced composites have become a promising alternative to synthetic fiber composites because of their favorable environmental characteristics, cost efficiency, and good mechanical properties. The present review provides a comprehensive examination of the manufacturing processes and mechanical properties of polymer composites reinforced with jute fibers. This study investigates the influence of several fabrication methods, such as hand lay-up, compression molding, injection molding, pultrusion, etc., on the mechanical properties of the composites. It also provides SWOT analyses of various manufacturing processes of jute fiber-reinforced composites. Important aspects, including fiber orientation, fiber/matrix adhesion, and the effects of different surface treatments on improving mechanical characteristics, such as tensile strength, flexural strength, and impact resistance, are discussed. The difficulties associated with moisture absorption, degradation, and the lack of uniformity in jute fibers, as well as approaches to alleviate these problems, are presented. The goal of this study is to establish a basis for future investigation and advancement in enhancing the mechanical properties of jute fiber-reinforced composites.

## 1. Introduction

Jute fiber is an organic fiber obtained from the bark of the jute plant, which is classified under the genus Corchorus. It is mainly cultivated in Bangladesh, India, and China. The Bangladesh Jute Knowledge Bank (https://bjkb.gov.bd/, accessed on 3 February 2025) provides a comprehensive overview of jute. It is a highly cost-effective natural fiber and ranks second in terms of production volume, just after cotton. Jute mostly consists of cellulose (60–70%), hemicellulose (12–14%), and lignin (5–10%) [1,2]. Due to its lustrous and smooth texture, it has been given the tag “the golden fiber”. Jute fiber is highly significant due to its renewable nature, capacity to decompose naturally, and positive impact on the environment. This makes it a viable and sustainable substitute for synthetic fibers [3]. Despite benefits like affordability, high strength-to-weight ratio, and good insulation properties, it has drawbacks, such as moisture absorption and quality variability [4,5,6].

Due to its numerous environmental, economic, and performance benefits, jute fiber is progressively being preferred over synthetic fibers, metals, and alloys. The main factor in selecting jute fiber is its sustainability. Contrary to synthetic fibers that come from non-renewable petroleum sources, jute fiber is renewable, biodegradable, and compostable. This characteristic of jute helps reduce its impact on the environment [7]. Furthermore, the manufacturing process of jute fiber requires considerably less energy and releases fewer greenhouse emissions in comparison to the production of synthetic fibers and metal processing [8]. From an economic standpoint, jute is a cost-effective option, offering a more affordable alternative to pricier synthetic fibers and metals. Affordability is essential for enterprises seeking to decrease material expenses while upholding quality and performance. Jute fibers have excellent performance characteristics, such as a high specific strength and stiffness, which make them well-suited for reinforcing polymer matrices in composite materials. These materials provide sufficient mechanical qualities that make them suitable for various applications, such as the automotive [9,10,11], construction [12,13,14], and packaging sectors [15,16]. In addition, jute fibers provide exceptional thermal and acoustic insulation characteristics, which enhance the functional capabilities of composites [6]. Jute fibers have exceptional resistance to corrosion and numerous chemicals, rendering them highly durable under diverse environmental situations [17]. The increasing favor for jute fiber over synthetic alternatives and metals in various industrial applications highlights the significance of research to develop jute fiber-based composites.

Jute fiber-reinforced composites (JFRCs) are materials in which jute fibers are incorporated into a polymer matrix to improve mechanical qualities. These composites utilize the robustness and rigidity of jute fibers while also taking advantage of the polymer matrix’s capacity to evenly distribute stresses and shield the fibers from harm caused by the environment [18]. Research and study of JFRCs are crucial because of the increasing need for sustainable and environmentally friendly materials. Utilizing natural fibers such as jute in polymer matrix composites presents a sustainable substitute for traditional glass or carbon fiber composites, hence diminishing the ecological impact of composite materials. Moreover, JFRCs offer a cost-efficient alternative that possesses adequate mechanical characteristics for a wide range of engineering uses [19]. The positive features and environmental benefits of jute fiber-based composites make them suitable for various sectors. JFRCs are utilized in the automobile sector to produce interior components, including door panels, dashboards, and seat backs. This application helps to decrease weight and enhance fuel efficiency [20,21]. JFRCs are commonly used in the construction industry to manufacture lightweight and long-lasting building materials such as partition boards, panels, and roofing sheets [5]. The furniture sector also utilizes JFRCs to produce visually appealing and eco-friendly furniture pieces. In addition, JFRCs are employed in packaging materials as a substitute for traditional plastics, aiding in the reduction of plastic waste [22]. The wide range of applications and increasing adoption of JFRCs highlight their significance and promise for further advancement.

The production method of jute fiber-reinforced composites plays a major role in defining their mechanical properties and overall performance. The choice of manufacturing procedures can have a substantial impact on the adhesion between fibers and matrix, the distribution of fibers, and the presence of voids in the composite. These factors directly influence the strength, stiffness, and durability of the material [23]. Comprehending and enhancing these procedures are crucial for creating top-notch JFRCs that fulfill certain application prerequisites. Processes such as resin transfer molding (RTM) and compression molding can enhance the mechanical characteristics of composites by improving fiber wetting and ensuring uniform resin distribution. Conversely, more straightforward techniques like hand lay-up might yield composites with reduced strength as a result of uneven distribution of fibers and increased presence of voids [24]. Hence, examining the manufacturing procedures aids in determining the most effective methods for fabricating JFRCs with exceptional mechanical characteristics.

Despite the abundance of studies on natural fiber composites, there is a notable gap in the research concerning different manufacturing processes for jute fiber-based composites and their effects on the mechanical properties of these composites. This work attempts to present a concentrated review of JFRCs, focusing on the distinct benefits and difficulties related to various manufacturing procedures. This paper aims to achieve two main objectives. Firstly, it seeks to investigate various manufacturing processes of JFRCs along with their strengths, weaknesses, opportunities, and threats. Secondly, it aims to analyze the mechanical properties, such as tensile strength, flexural strength, impact resistance, and fatigue performance, for each of these manufacturing processes. The study seeks to attain these objectives to offer a thorough comprehension of the correlation between manufacturing procedures and the resultant attributes of JFRCs. This will, in turn, provide guidance for future research and industry practices in the advancement of high-performance, sustainable composites. Thoroughly examining these subjects will not only enhance the current state of knowledge on JFRCs but also offer valuable insights into improving manufacturing procedures to produce high-quality composite materials.

## 2. Manufacturing Processes

For manufacturing jute fiber composite materials, the major steps are fiber preparation, matrix impregnation, molding, curing, etc. Fiber preparation varies depending on the state at which the fibers are used, e.g., roving, yarn, woven mat, chopped, randomly distributed, etc. The properties of the jute fiber-reinforced composites are highly dependent on these states during manufacturing. The methods of fiber treatment, resin impregnation process, molding technique, and curing condition greatly influence the properties of the manufactured composites [25]. The modification or cleaning of the fiber surface using alkaline solutions is usually performed during the fiber preparation [26,27,28,29]. The methods of matrix impregnation, molding, and curing are the most significant part of manufacturing fiber-reinforced composites. The oldest method of manufacturing fiber-reinforced composites is hand lay-up, which is also used for fabricating jute fiber-reinforced composites. Mostly, the composites are made using the hand lay-up method. However, with the advancement of technology, various easier processes of molding have been introduced like injection molding, compression molding, etc. Other processes, including kinetic mixing, pultrusion, etc., are also common [27]. The manufacturing processes of jute fiber-reinforced composites are discussed in the following sub-sections and for each manufacturing process. A SWOT analysis is presented for some manufacturing processes to help readers understand the strengths, weaknesses, opportunities, and threats involved in utilizing a particular manufacturing process for manufacturing jute fiber-reinforced composites.

### 2.1. Hand Lay-Up Method

**Figure 1:**
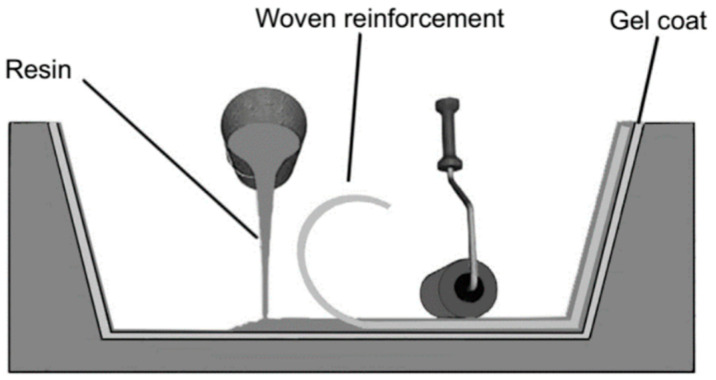
Hand lay-up schematic diagram [32,33].

Hand lay-up, as shown in Figure 1, is one of the oldest open-mold techniques for manufacturing natural fiber-reinforced composites [30]. Long and staple natural fiber-reinforced composite materials can be manufactured easily with the help of this method [31]. Wide variations in hand lay-up allow fibers to be oriented in a variety of ways, including unidirectional, inclined, or woven.

Due to its reduced directional dependence and tolerance to many forms of stress, hybrid composites are manufactured according to the procedure that is attracting attention from the composite manufacturing sector [34]. In this procedure, the mold surface is coated with an anti-adhesive chemical to prevent the composite from sticking to the mold and to facilitate its release [35]. Sometimes, a plastic sheet is inserted at the bottom and top portions of the mold plate to create a smooth surface [32]. The matrix material’s gel coat is applied to the lower mold surface, and the fiber is placed. Then, a roller applies a small amount of pressure to release any trapped air bubbles, and simultaneously, the matrix material is applied [36]. The solid product is removed from the mold cavity once the material is completely cured [37].

The hand lay-up procedure in composite manufacturing entails the physical placement of prepreg fibers onto a mold, relying on the skill and expertise of the operator [38]. One of the main challenges of this conventional technique is to achieve uniform pressure distribution across the surface and prevent the occurrence of flaws, such as wrinkles, when placing the material. To tackle these problems, researchers have adopted robotic lay-up systems to automate the process and enhance consistency and excellence in sectors such as aerospace and automotive [39,40]. In these works, an end-effector capable of replicating human movements during the hand lay-up of fibrous tissues or fabrics has been conceptualized, which can be used in the manufacturing of complex-shaped surfaces.

In most works, normal curing at room temperature was followed for the hand lay-up method. However, other processes can be used for a short time and are more effective in curing at higher temperatures. The curing stage of the process involves using techniques like UV-curable resin impregnation and curing with UV lamps [41] or using tools with heated surfaces to cure composite part lay-ups [42]. This stage ensures that the resin matrix is consolidated and hardened, resulting in the formation of a robust and long-lasting composite structure. In addition, employing methods such as wet-process hand lay-up molding of prepreg to eliminate bridging helps to maintain the integrity and quality of the end product [43]. Effective curing not only improves the mechanical strength and ability to be machined of the composite panel but also helps to decrease energy usage and increase the overall stability and appearance of hand lay-up products [44].

**Figure 2:**
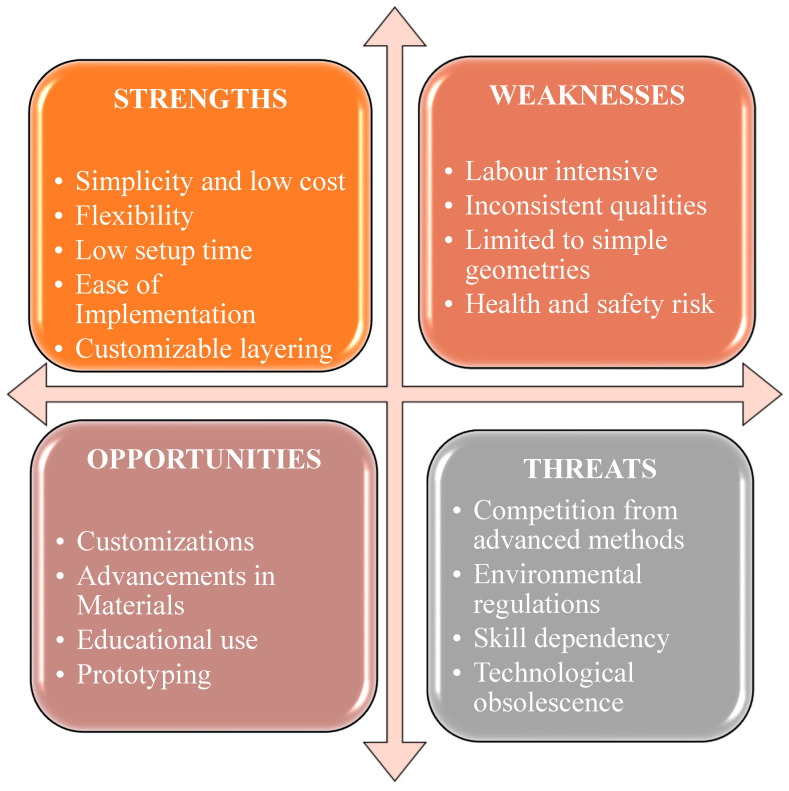
SWOT analysis of the hand lay-up process.

The SWOT analysis in Figure 2 highlights the hand lay-up technique’s strengths, including its simplicity, cost-effectiveness, and suitability for small-scale tasks and educational settings. It allows customization of layer arrangement, which can yield a polished surface when performed correctly [45]. Nevertheless, the procedure is demanding in terms of labor and time, therefore restricting both productivity and scalability. The inherent artisanal nature of the procedure may result in discrepancies in the end product, including uneven dispersion of fibers and resin concentration. Furthermore, it is most suitable for uncomplicated forms, therefore restricting its use in more intricate designs. Exposure to resins and solvents poses substantial health hazards, necessitating the implementation of appropriate protective measures [43].

Despite these challenges, the hand lay-up method has promising prospects for custom small-scale components, particularly in the aerospace, automotive, and marine industries. Advances in resins and fibers may enhance composite performance [46]. This technique is ideal for education and prototyping, allowing quick idea development before moving to advanced production. However, it faces competition from more automated methods like resin transfer molding (RTM) and automated fiber placement (AFP), which offer higher quality and efficiency. Environmental regulations and the need for skilled labor also pose challenges, potentially making hand lay-up less viable for large-scale industrial applications [47].

### 2.2. Injection Molding Method

**Figure 3:**
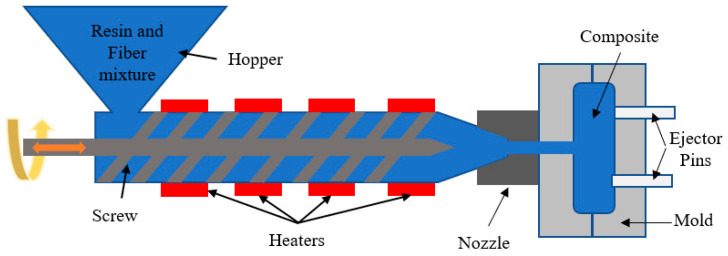
Injection molding schematic diagram.

When casting a complex shape, injection molding is a better option, even when manufacturing a natural fiber composite, as shown in Figure 3. Moreover, manufactured parts need less machining, which decreases labor costs. In this process, first, matrix materials like resins, hardeners, etc., are mixed and poured into a container named a hooper. Then, these are conveyed with the help of a screw and injected into the mold through a nozzle where fibers are already present. However, through conveying, sometimes heat needs to be applied according to the matrix type. If these are solid pallets, heat is necessary to melt them. After cooling, the composite is ready [48].

In this process, the appropriate fiber length is important so that the entire stress can be transferred from matrices to fiber, which can be found in Equation (1).

(1) $$L_{c}=\frac{\sigma_{uf}d}{2\tau }$$

where *L*_*c*_ = critical length of fiber;

*d* = fiber diameter;
*τ* = shear stress at the interface of fiber and matrix;
*σ_uf_* = ultimate tensile strength of fiber, respectively [32,49].

In this process, the interfaces of fibers and matrix face imperfect bonding. Therefore, maintaining the optimum length of fibers found from this equation is important [50].

**Figure 4:**
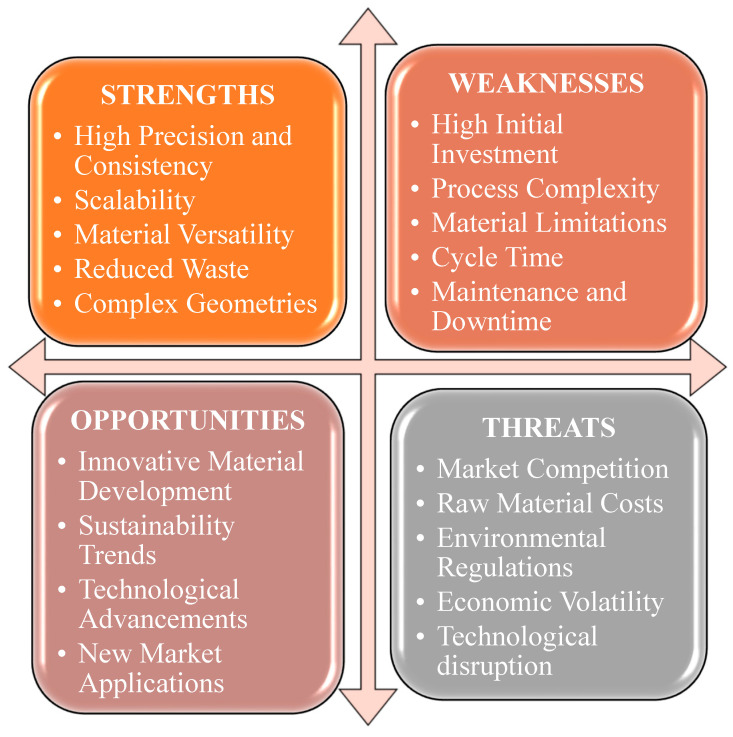
SWOT analysis of the injection molding process.

A SWOT analysis of the injection molding is shown in Figure 4. Injection molding is instrumental in enhancing the strength of jute composites by optimizing various process parameters. Research indicates that the mechanical properties of jute-reinforced composites can be significantly improved through specific injection molding techniques such as direct fiber feeding injection molding (DFFIM) [51]. By controlling temperature, pressure, and molding time, the mechanical performance of jute/polypropylene (jute/PP) composites can be improved [52]. Moreover, the incorporation of maleic anhydride grafted polypropylene serves to enhance the interfacial bonding between the jute fibers and the PP matrix [53]. Additionally, the application of Six Sigma methodology within the injection molding process allows for the identification and optimization of processing and material parameters [54]. Injection molding can be used to customize the strength of jute composites. Research has shown that the mechanical characteristics of composites reinforced with jute fibers can be tailored using different injection molding methods. Direct fiber feeding injection molding (DFFIM) [51], twin-screw extrusion, and injection molding [53] have been used to improve the bond between jute fibers and the polymer matrix. This has led to increased tensile strength and modulus of the composites. Furthermore, producing long jute fiber-reinforced polylactic acid (LJF/PLA) pellets for injection molding improves bending strength and stiffness [55]. Overall, controlling molding process parameters is important for achieving improved mechanical performance in jute-reinforced composites [52].

The injection molding process has several advantages, such as accuracy and uniformity, allowing for the manufacture of different parts with consistent quality and very few flaws. Its scalability makes it ideal for large-scale production, reducing costs. The process is highly adaptable, compatible with various composite materials, and allows for customizing material properties to meet specific needs. Additionally, it can minimize material waste, enhancing cost-effectiveness and environmental sustainability. It can create components with intricate details and complex geometries that are difficult to achieve with other methods [56].

However, this process has various weaknesses, such as higher initial investment in machines, molds, and tooling, posing challenges for small businesses. The complexity of the process demands skilled personnel and advanced equipment, leading to higher operational costs. Material limitations also exist, as some composites may struggle with high temperatures and durability, limiting their use in high-performance applications. Additionally, the process can be time-consuming, which can affect overall manufacturing efficiency. Regular maintenance of equipment and molds is required, and any downtime can lead to significant costs and disrupt production schedules [57].

Continual research to develop an injection molding process could yield novel composite materials with enhanced characteristics and various application scopes. Advancements in automation, process control, and mold design can improve efficiency, precision, and cost-effectiveness. This process can be used in various applications such as automotive, aerospace, healthcare, and consumer electronics to stimulate growth and enhance diversity [58]. However, the process is confronted with several threats despite the available prospects. The presence of competitive production methods and materials, such as additive manufacturing and traditional metalworking, may potentially restrict the market share. Volatility in the price of primary resources, such as fibers and polymers, can have a significant influence on the profitability and pricing tactics of a business. The implementation of more rigorous environmental rules for industrial processes and material disposal may result in higher expenses and operational difficulties. Fluctuations in the economy and volatility in the global market might impact the demand for composite products and the investment in manufacturing facilities. Ultimately, the rapid progress in alternative manufacturing technologies, such as 3D printing, may potentially endanger the conventional injection molding business by providing more adaptable and economical options [59,60,61].

### 2.3. Compression Molding Method

**Figure 5:**
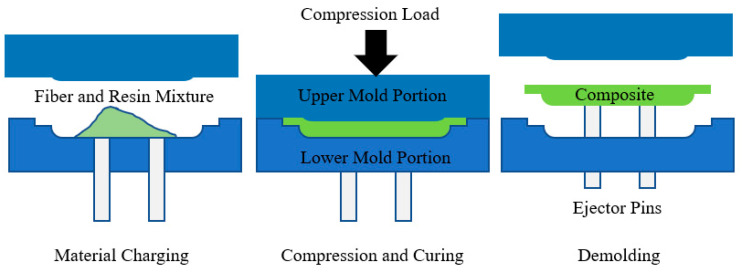
Compression molding schematic diagram.

Compression molding is very useful for manufacturing both thermoplastic and thermosetting composites made of natural fibers. This process is quite popular in industry [62]. In this process, there are two mold sections: upper and lower, as shown in Figure 5. The fibers and the matrix are loaded in the lower section of the mold and the upper section is pressed with appropriate pressure and temperature during the molding process to achieve the desired shape inside the mold cavity. The combined autoclave and hot press process is known as the compression molding process. This method can deal with both short and long fibers. In the autoclave process, the reinforcing fibers of thermoplastic material are placed in a certain order on the mold. The laminate is then sealed in a negative pressure bag and placed in the autoclave. After going through a heat and pressure cycle, the laminate is cured, and the desired composite is made [63]. In the hot press method, however, the mold does not need to be closed. A certain amount of natural fibers are piled and placed in the cavity within a tight mold [32,64].

Compression molding is used to improve the strength of jute fiber composites by optimizing process parameters. Research has shown that the mechanical characteristics of jute fiber-reinforced polypropylene composites can be greatly improved by optimizing multiple parameters during the compression molding process [52]. The incorporation of long, intermittent fiber platelets and continuous fiber preforms in compression molding has demonstrated significant enhancements in strength. This emphasizes the possibility of enhanced structural features and long-lasting quality in various uses [65]. Additionally, the production of hybrid composites using epoxy-based glass and jute fibers has been improved by employing a compression molding process. This process includes altering the arrangement of the fibers, which results in improved tensile strength, flexural strength, and resistance to water absorption [66]. Multiple studies have confirmed the effectiveness of compression molding in producing JFRCs with improved mechanical and structural properties [67,68,69].

This process has various advantages, such as superior mechanical performance, cost efficiency, flexibility in material selection, waste reduction, and consistent product quality. This procedure is highly effective in forming robust and long-lasting composites by optimizing parameters and utilizing a range of fiber types and matrices. However, this process has several drawbacks, including extended cycle durations, restrictions in manufacturing intricate shapes, demanding high-pressure conditions, necessitating additional post-processing procedures, and encountering difficulties in working with materials such as long fibers [70,71].

**Figure 6:**
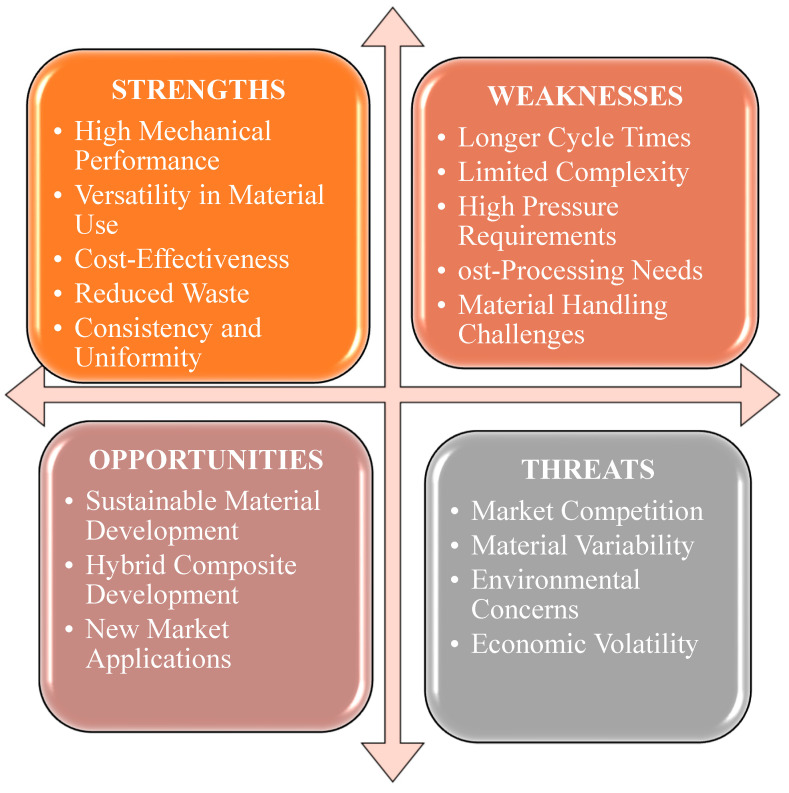
SWOT analysis of the compression molding process.

The increasing need for sustainable materials, technological progress, the broadening range of market uses, and the emergence of hybrid composites are the main opportunities for this process. These characteristics can optimize process efficiency, minimize costs, and create new opportunities for use in diverse sectors. However, various dangers could potentially hinder the efficiency of compression molding. The factors encompassed in this list are material unpredictability, supply chain disruptions, technology obstacles, and environmental effect concerns. To optimize the use of compression molding for composite fabrication, it is important to address these threats and take advantage of the strengths and possibilities [72,73]. A SWOT analysis of this process is summarized in Figure 6.

### 2.4. Resin Transfer Molding Method

**Figure 7:**
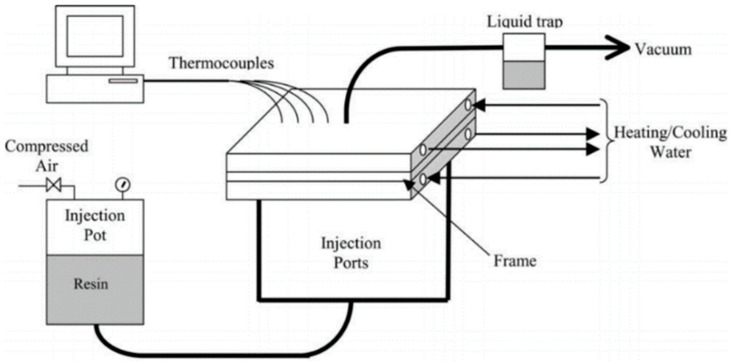
Resin transfer molding schematic diagram [32,74].

For thermosetting composites, the resin transfer molding method, which is a verified type of injection molding, shown in Figure 7, is a good option. Normally, this process is used to deal with long fibers or woven fibers [74]. Various criteria like injection pressure, temperature, fiber structure, resin viscosity, fiber mat permeability, and mold configuration are important to note in this process. This method supports production on a large scale, which is relatively cost-effective compared to other methods [50]. The requirement of lower temperature and abstinence from thermomechanical degradation makes this process a better choice than many other methods. However, natural fibers are less compact than glass fibers, which results in natural fiber composites having a lower density in this process [75].

RTM is similar to injection molding. In this process, to let fibers deform, a small clearance needs to be maintained between mold edges. The velocity difference is greater at the start of the injection procedure and decreases as the time difference increases. This velocity differential is decreased by the flow resistance [76]. Utilizing numerous injection gates, resin flow can be accelerated without raising the injection pressure. However, many gates make the process more complicated and result in a high number of bubbles at the meeting point of flow fronts. This empty content area significantly lowers the mechanical characteristics. The injection pot and mold must remain under vacuum before beginning the injection process to minimize the voids in the final product [32]. Additionally, a higher flow resistance obstructs the flow path, causing the flow to enter a channel with lower resistance to apply injection pressure, which escalates the effect. As a result, the amount of time needed at the bottom’s edge flow increases, which has a negative impact on format spillage and dry areas [77]. The local velocity field might vary from point to point at a microscopic scale despite the average velocity field of resin flow being smooth. Local velocity field roughness is primarily caused by local capillary pressure, permeability, and non-uniform microstructures [78].

**Figure 8:**
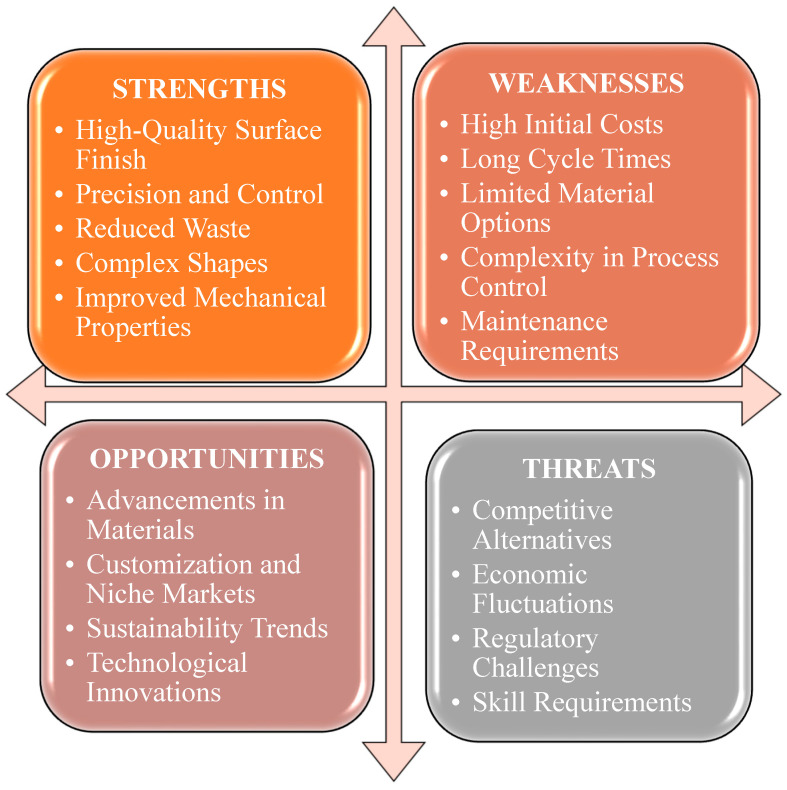
SWOT analysis of resin transfer molding.

A SWOT analysis of the resin transfer molding is shown in Figure 8**.** This process significantly improves the mechanical properties of jute fiber composites. Studies indicate enhancements in tensile strength and modulus for jute fiber-reinforced composites using RTM. Research on jute/PLA composites shows that surface treatments like NaOH and silane coupling agents, when combined with RTM, notably boost tensile performance [51]. Moreover, a comparison between polyester and vinyl ester resins in jute/aramid hybrid composites fabricated by RTM revealed that polyester resin exhibited significantly greater tensile strength and microhardness [79]. In addition, optimizing fiber size and percentage can further enhance the strength of jute-based composites, as shown in the study on jute fiber-reinforced polypropylene composites [80].

The advantages of RTM include its capacity to generate superior surface finishes and meticulous control over resin flow and fiber positioning, resulting in consistent product quality. The procedure additionally reduces resin waste and is capable of handling intricate geometries, hence enhancing the mechanical qualities of the composites. Nevertheless, RTM exhibits many limitations, including high upfront costs for molds and equipment, which can be a barrier for smaller businesses. The process often involves longer cycle times and is primarily limited to thermosetting resins, restricting material options [81,82].

RTM benefits from developments in materials and technology, which can improve its uses and efficiency. The increasing demand for sophisticated composites in the aerospace and automotive sectors, along with a shift toward sustainability, creates favorable conditions for RTM. Automation advancements can enhance process efficiency and reduce costs, while tailored solutions meet the specific needs of specialized markets. However, RTM faces competition from alternatives that may offer lower costs or faster production times. Economic fluctuations and regulatory constraints can affect its cost-effectiveness and compliance. Additionally, the need for skilled operators and potential market oversaturation may pose challenges to profitability and operational effectiveness [83,84].

### 2.5. Pultrusion Method

**Figure 9:**
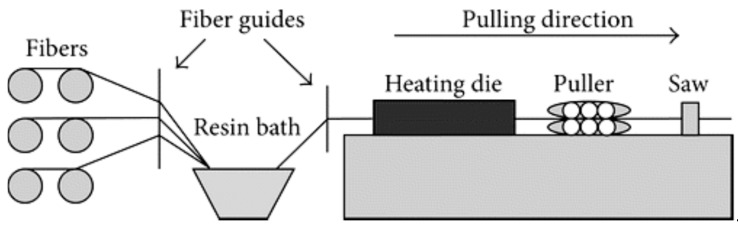
Pultrusion process schematic diagram [86].

Pultrusion is a straightforward procedure for manufacturing composite materials with a constant cross-sectional area, as shown in Figure 9. Since this process uses a continuous processing method, the process has a low labor content and a high raw material conversion efficiency. Because of their consistent quality, there is very little necessity for further finishing activities before using the pultruded items [85]. Several factors need to be considered, such as the mutual interactions between heat transfer, resin flow, and cure reaction, variation in the material properties, and stress evolutions. These affect the process advancement together with the mechanical properties and the geometrical accuracy of the final product [86].

In this process, an exothermic reaction takes place; therefore, it is slightly difficult to handle [87]. In this process, various types of fibers can be used, such as roving, woven, etc. First, fibers are pulled out with the help of a guide and soaked with matrixes. This soaking can be performed in an open bath or a resin injection chamber. Then, the soaked fibers are pulled out with the help of a pulling mechanism through the heating die [88]. The temperature in the center of the soaked die is less than that of the side due to low thermal conductivity. That is why the heating process should take enough time. Various catalysts or electric heaters can be used for that reason [87]. Consequently, the liquid portion turns to gel then on the verge of being solid as a result of the chemical reaction caused by the catalyst. Due to these reactions, the matrix shrinks, which develops the property. Finally, after solidification, the composite is pulled out and cut into the desired shape [86,89].

**Figure 10:**
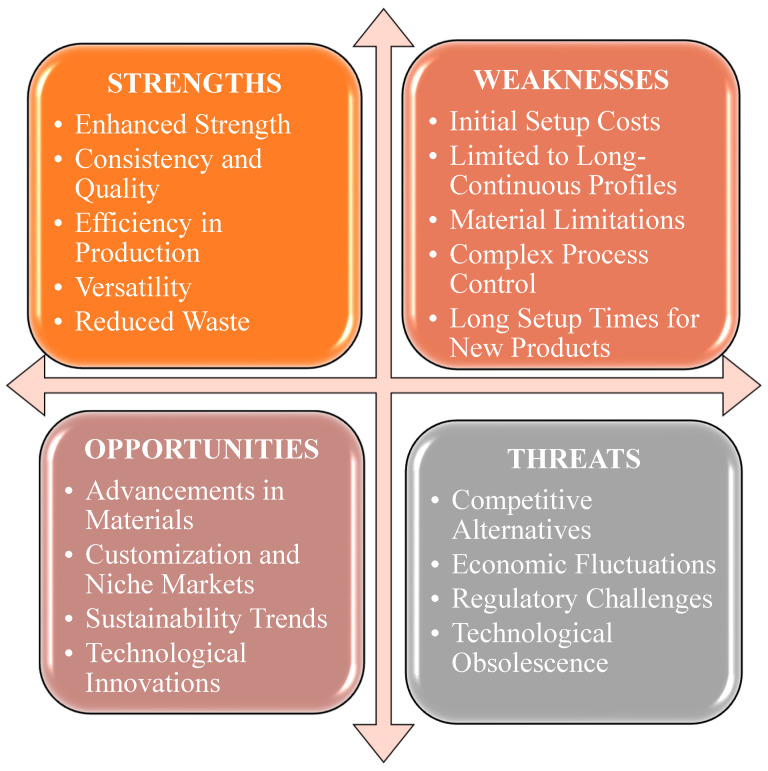
SWOT analysis of the pultrusion process.

Pultrusion significantly enhances the strength of composite materials made from jute, as mentioned in Figure 10. Investigation into the process of thermoplastic pultrusion demonstrates that fine-tuning molding parameters, such as the speed at which the material is pulled and the temperature at which it is processed, has a beneficial impact on the mechanical characteristics of the material, but only up to a certain limit. Past this threshold, the qualities of the materials may deteriorate as a result of factors such as the formation of empty spaces and the breakdown of fibers [90]. Incorporating zinc oxide filler in jute/epoxy composites through compression molding improves mechanical strength. The highest improvement is seen when using 25% filler content, suggesting stronger bonding between the fibers and the matrix [91]. Additionally, the use of pultruding jute fabrics with a polymeric matrix enhances the bonding between natural fibers and cement, resulting in heightened strength, resilience, flexural strength, and bending stiffness of fiber cement sheets [92]. Using the pultrusion method for jute fiber-reinforced polyester composites with hybrid fillers results in a significant tensile strength increase, reaching an optimal 73.14 MPa with the ideal filler composition [93].

The pultrusion method can improve the mechanical characteristics of composites, such as tensile strength and stiffness, which makes it well-suited for structural applications. The precise control over resin impregnation and fiber alignment ensures excellent quality and homogeneity in this process. It is a very efficient process that allows the manufacturing of long, continuous profiles at high rates. This system allows for the use of different types of fibers and resins and it reduces waste by applying resin with precision. However, this process needs an initial high investment that inhibits smaller production. The process is suited for long, uniform profiles and may not be ideal for complex designs. There material options to choose from in this process are limited to specific resin systems and fiber types, and achieving optimal process parameters is challenging. It requires considerable setup time to develop new products, impacting production adaptability [94,95,96].

Improved performance and an expanded range of applications are the main opportunities of this process. The increasing demand for composites in construction, automotive, and aerospace offers growth opportunities. Technological advancements in automation and process optimization have the potential to enhance efficiency and decrease the expense of this process. The use of natural fibers and recyclable resins is well-suited for the pultrusion process. This technique is capable of meeting the demands of specialized markets by generating customized profiles and hybrid composites. However, it faces competition from alternative processes like resin transfer molding and filament winding, which may offer greater versatility or lower costs. Economic volatility and fluctuating raw material prices can affect cost-effectiveness, while regulatory challenges may increase complexity and expenses. Rapid developments in alternative composite technologies could outpace improvements in pultrusion, and market saturation may lead to heightened competition, downward pressure on prices, and decreased profit margins [97,98].

### 2.6. Vacuum Molding Method

**Figure 11:**
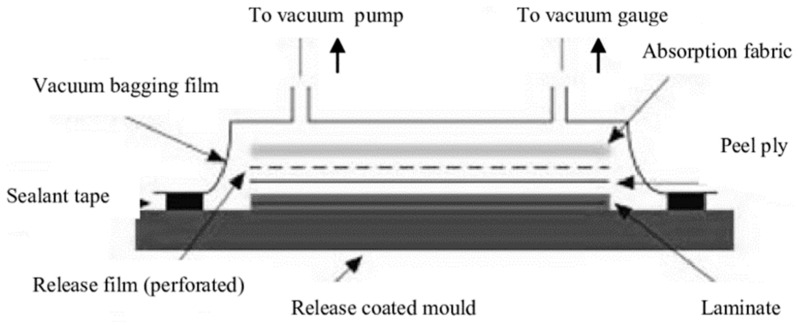
A vacuum molding process schematic diagram [102].

A classic and cost-effective process, vacuum molding, as shown in Figure 11, is broadly used in industrial applications. The speed and efficiency of repetition make this process popular; however, it has a huge drawback of not having consistency of thickness throughout [99,100], and the finishing of open surfaces is not very good [101].

This process has four stages: lay-up, pre-filling, filling, and post-filling [103,104]. During the lay-up stage, a fabric preform is placed on the mold’s solid side and covered with peel-ply for easy separation, as shown in Figure 11. A distribution medium may be added over the peel-ply to enhance resin flow. After installing the resin inlet and vacuum vent tubes, the mold is closed with a vacuum bag sealed with tape. At the pre-filling stage, the preform is compacted after the cavity has been sealed and the inlet has been clamped. The inlet is opened at the end of the pre-filling cycle, allowing the resin to seep into the preform. The pressure inside the cavity changes throughout the filling stage. The inlet is typically clamped once the resin flow front reaches the preform’s end to stop the resin from flowing into the cavity. During the post-filling stage, excess resin is removed to balance laminate thickness and resin pressure. After proper curing, the composites are released from the mold [100].

Vacuum-assisted resin transfer molding (VARTM) is used to improve the strength of jute composites. By utilizing double-bag air cushioning [105], vacuum degassing [106], and sophisticated VARTM procedures with pressure control [107], the performance of resin infusion is enhanced, resulting in less void content and enhanced mechanical characteristics. Studies have shown that fine-tuning the vacuum infusion parameters, such as supply pressure and soaking time, significantly enhances the flexural strength and modulus of jute composites [107]. The incorporation of short jute fibers into epoxy resin composites by vacuum infusion has been shown to enhance tensile strength, particularly when the fibers are aligned in the direction of the applied force [108].

### 2.7. Autoclave Molding Method

**Figure 12:**
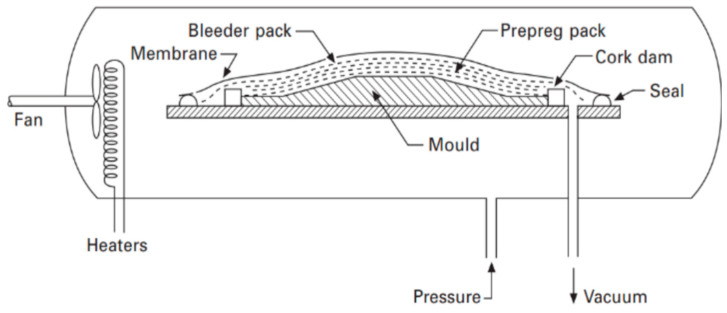
Autoclave molding process schematic diagram [32,109].

To create high-value composites from prepregs, autoclave molding is widely applied, especially in the aerospace sector. High-quality, consistent moldings may be manufactured in this process. However, the procedure is labor and capital-intensive [109]. In this process, a membrane, known as the bag, separates the laminate from the autoclave’s interior, with a pressure line connecting the enclosed area to the outside, as shown in Figure 12. A porous membrane inside the bag ensures constant gas flow. A porous release layer is placed over the prepreg laminate on the molding tool, allowing for resin absorption. The laminate typically has release layers and absorbers on both sides, with a dam around the edges to prevent rounding from bag tension. A top plate may be added to the exterior of flat laminates for a more uniform finish. The final laminate’s quality will be affected by two of these components, in particular, the prepreg and absorber [110].

The pressure and temperature within the autoclave are then independently adjusted to give even control of pressure throughout the surface and to thermally control the cure before a vacuum is drawn inside the cover membrane, which is needed to eliminate volatiles and porosity. Dry laminates, which often result from applying pressure too early and forcing out low viscosity matrix, and porous laminates, which result from applying pressure too late with high viscosity cured resins, can both be avoided by optimizing the application of pressure and vacuum [32,109].

### 2.8. Extrusion Molding Method

**Figure 13:**
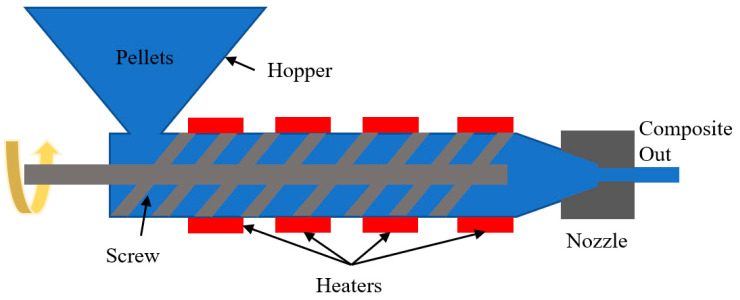
Schematic diagram of extrusion molding.

Extrusion molding is a similar process to injection molding except for the fact that extrusion is semi-restrictive molding by an extrusion die, whereas injection molding is extremely restrictive molding by an injection mold. Here, like injection molding, a hopper is used to feed pelletized resin into an extruder [111]. The material is then pushed through a die. A screw continuously extrudes resin through a mold to create a molded object, as shown in Figure 13.

## 3. Mechanical Properties

### 3.1. Hand Lay-Up Process

#### 3.1.1. Tensile Properties

The tensile properties of jute fiber-reinforced composite materials have been studied by various researchers from many aspects, as mentioned in Table 1. Gopinath et al. [25] tested jute fiber composites that were reinforced in two different resins, epoxy and polyester, and two different treated fibers were used, by 5% and by 10% NaOH, while the fiber-to-resin weight ratio was 18:82. They showed that the epoxy composite had better mechanical properties than that of polyester and that the 5% NaOH-treated fiber composite had better tensile properties than that of the 10% one. Hand lay-up molding was also studied by Mishra and Biswas [112], with 12%, 24%, 36%, and 48% wt. jute reinforced in epoxy, which showed that an increment in fiber loading decreased the void fraction as well as increased the tensile properties and other properties. This technique was also used by Venkateshwaran and Perumal [113] for reinforcing woven jute in epoxy and compared with banana/jute hybrid composites, where it was shown that the banana/jute/banana composite performed better in terms of tensile properties. Sen and Reddy [114] used heat-treated main and cross-directional woven textile jute mats in epoxy. With the help of the hand lay-up process, they reinforced a concrete-reinforced concrete (RC) beam and reported a noteworthy enhancement in the load-bearing capacity of the RC beam. Chandramohan et al. [115] used this method to reinforce a 5-layer fabric of continuous jute fiber (50%) in polyester resin and compared it to hybrid fiber composites. Das and Bhowmick [116] prepared jute fiber reed and jute silver from raw jute with the help of 30% jute batching oil in water emulsion and then reinforced unsaturated polyester resin using the hand lay-up technique and compared them. They showed that the tensile properties of the jute fiber reed composite were higher than those of jute silver; as well, the increment in fiber weight percentage had a positive effect on tensile properties. Manik et al. [117] reinforced jute fiber (70% wt.) in epoxy using the hand lay-up method and compared it with a coconut and human hair composite; the jute composite showed better tensile properties than the other two composites. Deieu et al. [118] reinforced 0.4% NaOH-treated jute fiber in polypropylene using the hand lay-up method and compared it with the same composite but a compatibilizer as coupling agent, maleic anhydride polypropylene (MAPP), was used, which showed that MAPP had a positive effect on tensile properties. With fiber loading at 8%, 10%, and 12% wt. or, respectively, 7%, 8.5%, and 10% vol., Wang et al. [119] reinforced untreated and chemically treated long, unidirectional jute fibers in epoxy using the hand lay-up technique and showed that an increment in fiber loading increased the tensile properties and that chemical treatment of fibers had a positive effect on those properties. Bambach [120] compared biaxial woven jute fabric (44.3 ± 2.2% in vol.)-reinforced epoxy composite using the hand lay-up technique with other natural fiber composites in terms of properties and found that the increment in fiber layers had a positive effect.

The hand lay-up molding technique with low pressure was applied by Dobah et al. [121] for reinforcing polyester using novel woven jute fabric plies (25% in volume). They reported a higher tensile strength when the samples were tested in the uniaxial direction compared to the multi-axial direction. The hand lay-up technique followed by light compression molding was used by Boopalan et al. [122] for reinforcing epoxy with cross-plied jute fibers and compared with banana/jute hybrid fiber composites in different compositions in terms of tensile properties; the 50:50 jute and banana fiber turned out to be the best. Singh et al. [123] used combed unidirectional untreated and treated with 5% NaOH jute fibers (40% vol) in epoxy using hand lay-up followed by the compression molding method, and the results showed that surface treatment increased tensile strength. They also reported that the jute composite showed higher tensile strength than other fiber composites used. Untreated and 20% NaOH-treated jute mat were used as reinforcement in epoxy by Boopalan et al. [124] with the help of hand lay-up followed by light compression molding and compared to a sisal composite in terms of tensile properties. The treated jute fiber composite showed better properties compared to the treated sisal fiber-reinforced composite, as well as the untreated jute and sisal fiber composite.

The hand lay-up technique with the help of a compression molding machine was used by Mache et al. [125] to prepare bi-directional untreated woven jute fiber-reinforced polyester composite to make squared and double-hat-shaped sectioned specimens with 3- and 4-plies and compared them with 4-ply glass composite in terms of tensile properties. The glass showed better performance but the increment in jute fiber volume fraction had a positive effect on properties. Cavalcanti et al. [126] also used a hand lay-up technique with a hydraulic press to reinforce epoxy using twine form fabric weaved in a bi-directional mat of untreated, treated with NaOH, and mixed-treated (alkalized + silanized) jute having 30% volume fraction and compared it with hybrid fiber composites in terms of mechanical properties, and showed different effects of treatment on various hybridization, such as chemical treatments having a positive effect on the jute/sisal composite but a negative effect on the jute/curaua composite. Joseph et al. [127] used untreated and alkali-treated jute fabric in epoxy with the hand lay-up process used for composite preparation and compression molding for the curing process and showed that alkali treatment had a positive effect on tensile properties. Using the hand lay-up technique followed by static compression, Gupta [128] used jute fiber (16% wt.) in polyester. Fibers were 5% NaOH-treated, polylactic acid-coated, and treated with both, which showed that the treated fiber composite had higher tensile properties than raw fibers, and alkali-treated and PLA-coated ones had the highest values. Pawar et al. [129] used 5% wt. NaOH-treated matted jute (10–50% wt.) in epoxy using the hand lay-up technique followed by static compression, which showed a positive effect of fiber increment on tensile properties, but when adding granite powder as a filler, the filler content increment had a negative impact on the property. Singh et al. [130] used the hand lay-up technique followed by hot pressing to reinforce long jute fiber in epoxy, which showed that an increment in the fiber layer increased tensile properties, but compared with hemp composite, hemp had better properties. Using the hand lay-up technique followed by a hydraulic hot press, Kakati et al. [131] used non-woven/fabric jute in a mixture of soy flour-based resin, unsaturated polyester resin, and *R. heudelotii* oil-based alkyd resin, and showed that the increment in alkyd resin increased the values of the tensile properties.

#### 3.1.2. Compressive Properties

The compressive properties of jute fiber-reinforced composites have not been much studied. The reason for this may be the complexity of the test setup and the insignificance of compressive properties in the application sectors. The compressive properties of jute fiber-based composites fabricated using the hand lay-up method are mentioned in Table 2. Bambach et al. [120] studied biaxial woven jute fabric (44.3 ± 2.2% in vol.) in epoxy resin using the hand lay-up technique compared with other natural fiber composites in terms of properties and found that the increment in fiber layers had a positive effect on properties. Untreated, 4%, 5%, and 7% NaOH-treated woven jute fabrics (25%wt.) were utilized by Kabir et al. [132] in unsaturated polyester resin applying the hand lay-up method, which showed an increment in fiber matrix bonding with the increment in the percentage of NaOH concentration, which resulted in an increment of compression properties. By using the hand lay-up technique with the help of a compression molding machine, Mache et al. [125] studied bi-directional untreated woven jute in polyester to make squared and double-hat-shaped sectioned specimens with 3- and 4-plies and compared the outcomes to 4-ply glass composite in terms of compressive properties, with the glass composite outperforming the jute fiber in terms of performance but the increment in jute fiber volume fraction had a negative effect on properties.

#### 3.1.3. Flexural Properties

Table 3 shows the flexural properties of jute-based composites manufactured by the hand lay-up method. Jute fiber treated with NaOH was used in epoxy and polyester by Gopinath et al. [25] with the help of the hand lay-up method, keeping the fiber/resin weight percentage ratio at 18:82. The results showed that the 5% treated jute composite had better mechanical properties, such as tensile properties, than that of the 10% one, but in terms of resin used, the polyester composite had better flexural properties than that of the epoxy one. Kabir et al. [132] studied untreated, 4%, 5%, and 7% NaOH-treated woven jute fabric (25% wt.) in unsaturated polyester resin using the hand lay-up method, which showed an increment in flexural properties with the increment in the percentage of NaOH concentration. Mishra and Biswas [112] used the hand lay-up method to reinforce epoxy using a bi-directional jute fiber mat (12%, 24, 36%, 48% wt.), and results showed that when fiber loading increased, the void fraction dropped and the flexural properties and other parameters were enhanced. Using a similar technique, Venkateshwaran and Elaya [113] studied woven jute fiber-reinforced epoxy composites and compared them with hybrid banana/jute composites. The banana/jute/banana composite showed better flexural properties. Chandramohan et al. [115] studied a five-layer continuous jute fabric (50%) composite made of polyester resin and compared it to hybrid fiber composites using bamboo fibers. They reported that the hybridization of jute fiber with bamboo improved the physical and mechanical properties of the composite and made them compatible with glass fiber-reinforced composites. Manik et al. [117] used the hand lay-up method to study jute fiber (70% wt.) in epoxy and compared it with a coconut and human hair composite, which showed that the jute composite had better flexural properties than the other two. With the help of 30% jute batching oil in water emulsion, Das and Bhowmick [116] prepared jute fiber reed and jute silver from raw jute, which was used in unsaturated polyester resin using the hand lay-up technique to manufacture composites and compared them with each other. The result showed better flexural properties of the jute fiber reed composite than those of jute silver ones and the increment in fiber weight percentage had increased flexural properties. By using the hand lay-up method, using 0.4% NaOH-treated jute fiber in polypropylene and comparing it to the same composite but using a compatibilizer MAPP as the coupling agent, Deieu et al. [118] demonstrated that MAPP had a positive impact on the flexural characteristics.

Boopalan et al. [122] compared cross-plied jute fiber-based epoxy composites with banana/jute hybrid fiber composites in different compositions in terms of tensile qualities and found that the ratio of 50:50 in weight of jute and banana fiber was the best. Singh et al. [123] studied combed unidirectional untreated and treated with 5% NaOH jute fibers (40% in volume) in epoxy, which revealed that applying a surface treatment to a composite made from jute increased its flexural strength and that the jute composite showed higher flexural strength than other fiber composites. Boopalan et al. [124] studied untreated and 20% NaOH-treated jute mats in epoxy using hand lay-up followed by light compression molding and compared them with sisal composite in terms of flexural properties. They showed that the treated fiber composite showed better properties, but the sisal fiber composite outperformed the jute ones. The hand lay-up technique with a hydraulic press was employed by Cavalcanti et al. [126] to manufacture composites using a bi-directional mat of untreated, treated with NaOH, and mixed-treated (alkalized + salinized) jute with a 30% volume fraction in an epoxy matrix. The flexural properties of the composites compared to those of hybrid fiber composites revealed various treatment effects on various hybridizations, such as alkali treatments having a positive effect on jute/curaua composite but a negative effect on jute/sisal composite, whereas mixed treatments showed a positive effect on jute/curaua but a negative effect on jute/sisal composite. Joseph et al. [127] used untreated and alkali-treated jute fabric in epoxy for composite preparation and compression molding for the curing process and showed that the alkali-treated fiber composite had better flexural properties. Gupta [128] studied polyester with jute fibers (16% wt.) that were 5% NaOH-treated, polylactic acid-coated, or treated with both and showed that treated fiber composites had higher flexural properties than raw fibers, with alkali-treated and PLA-coated ones having the greatest values. Pawar et al. [129] investigated 5% wt. NaOH-treated matted jute (10–50% wt.) in epoxy and showed a positive effect of fiber increment on flexural properties, and when adding granite powder as filler, the filler had a positive impact on properties, but the filler content increment had a negative impact on the property. Singh et al. [130] reinforced long jute fiber in epoxy, which showed that the fiber layer increment had a positive effect on flexural properties when compared with hemp composite, but hemp showed higher values. Kakati et al. [131] used non-woven/fabric jute in a mixture of soy flour-based resin, unsaturated polyester resin, and *R. heudelotii* oil-based alkyd resin, which demonstrated that the increment in alkyd resin increased flexural properties.

#### 3.1.4. Impact Properties

The impact properties of jute fiber-reinforced composites using the hand lay-up method are summarized in Table 4. Gopinath et al. [25] studied NaOH-treated jute fibers in epoxy and polyester resin with a weight percentage ratio of 18:82 for Charpy impact strength, which showed that the polyester composite had a greater impact strength than epoxy one, whereas 5% treated jute composite had a better impact strength than 10%. However, neither was relatively high, like the values for metal. Mishra and Biswas [112] investigated bi-directional jute fiber mat (12.4%, 24.4%, 36.6%, 48% wt.) in epoxy, which showed an increment in fiber weight percentage increased the impact strength because the higher the fiber volume, the more energy required to break the interlaced fiber bundles. Venkateshwaran and ElayaPerumal [113] studied woven jute in epoxy and compared it with a jute/banana hybrid composite. The jute/banana/jute showed better impact strength. Chandramohan et al. [115] studied a five-layer continuous jute cloth (50%) in polyester resin and compared it to hybrid fiber composites and reported that the wet composite showed higher impact strength. Manik et al. [117] investigated 70% wt. jute fiber in epoxy and compared it with a coconut and human hair composite with the help of the Charpy and Izod impact test, which showed that the jute composite had higher impact properties than the other two composites in both test cases. Deieu et al. [118] studied 0.4% NaOH-treated jute fiber in polypropylene and compared it with the same composite but a compatibilizer as coupling agent, maleic anhydride polypropylene (MAPP), was used with the Charpy impact test, which showed that MAPP had hardly any effect on impact strength. Boopalan et al. [122] used cross-plied jute fibers in epoxy and compared them to banana/jute hybrid fiber composites in various ratios. The Izod impact test result showed that 50:50 jute and banana fiber performed the best. Singh et al. [123] used combed unidirectional untreated and 5% NaOH-treated jute fibers (40% in volume) in epoxy and showed a negative impact of treatment, while untreated jute composite had a higher impact strength, but it was lower than other fiber composites used if treated. Cavalcanti et al. [126] investigated twine form fabric weaved in a bi-directional mat of untreated, treated with NaOH-, and mixed-treated (alkalized + silanized) jute with a 30% volume fraction in epoxy matrix and the flexural properties of the composites were compared to those of hybrid fiber composites, which revealed that alkali treatments had a positive effect on jute/curaua composite but a negative effect on jute/sisal composite, whereas mixed treatments showed a positive effect on jute/curaua but a negative effect on jute/sisal composite. Joseph et al. [127] utilized untreated and alkali-treated jute fabric in epoxy for composite preparation and Izod impact testing was conducted. They demonstrated that the alkali-treated fiber composite showed better impact strength. Gupta [128] used jute fiber (16% wt.) in polyester, and the fibers were 5% NaOH-treated, polylactic acid-coated, and treated with both. The Izod impact test revealed that the raw jute composite had higher impact strength than alkali-treated as well as PLA-coated but lower than alkali-treated and PLA-coated. Pawar et al. [129] studied 5% wt. NaOH-treated matted jute (10–50% wt.) in epoxy, which showed a positive effect of fiber increment on impact strength. The incorporation of granite powder as filler had a positive impact overall.

#### 3.1.5. Hardness

The hardness of jute fiber-reinforced composites has not been studied much, and most of the work has reported the Rockwell hardness value. Gopinath et al. [25] studied Rockwell hardness and reported that 5% NaOH-treated fiber composite had a greater hardness value than that of 10%, but no significant difference was observed between polyester and epoxy resins (see Table 5). Mishra and Biswas [112] showed that the increment in fiber weight percentage resulted in an increment in hardness because of higher fiber content. Manik et al. [117] investigated jute fiber-reinforced epoxy composite and observed that the Rockwell hardness of jute fiber-reinforced composite was greater than coconut fiber composites but lower than human hair-reinforced composites. Kakati et al. [131] developed non-woven/fabric jute-based composites using a hand lay-up technique followed by a hydraulic hot press. The matrices of the composites were soy flour-based resin, unsaturated polyester resin, and *R. heudelotii* oil-based alkyd resin. The results showed that the higher the alkyd resin percentage, the more hardness value achieved.

### 3.2. Vacuum Molding

#### 3.2.1. Tensile, Flexural, and Impact Properties

The vacuum molding method is rarely used for manufacturing jute fiber-reinforced composites. The tensile and flexural properties of such composites are summarized in Table 6. Biswas et al. [133] studied unidirectional jute fiber (52% wt.) in epoxy, and the composite was compared with bamboo. The results showed that bamboo-based composites had greater tensile and flexural properties compared to jute-based composites. Rodriguez et al. [134] investigated untreated and 5% NaOH-treated bi-directional woven jute fabric in epoxy vinylester resin and compared the flexural strength of those composites with each other. The results showed that the untreated composite had better flexural properties than that of the treated one. They also showed that the untreated composite exhibited better impact energy than the treated one, which was 56.5 ± 2.4 J/m and 47.2 ± 4.2 J/m, respectively. They reported that fiber damage due to long time exposure is the main reason for the ineffectiveness of fiber treatment in the development of strength.

### 3.3. Heating in Hollow Cylindrical Glass

Using hollow glass cylinder as a mold, Ray et al. [135] studied untreated and 5% NaOH-treated white jute fibers (35% vol.) in vinylester while heating was applied. The results showed that alkali treatment causes improvement in flexural properties (Table 7), but treating the fibers for a long time causes degradation in the fiber and results in lower flexural properties. They also reported that fiber treatment caused a decrease in the Charpy impact strength of the composites, as shown in Table 7.

### 3.4. Extrusion Method

Limited studies have been found in the literature on the use of extrusion processes to manufacture jute fiber composites. Cabral et al. [136] studied uniaxial jute yarn with various volume fractions in polypropylene to manufacture composites using the extrusion method. They showed that an increment in volume fraction had a positive effect on tensile properties, as summarized in Table 8. In terms of Izod impact strength, up to 18% volume of fiber, the composite impact strength was increased but adding more fibers decreased the strength.

### 3.5. Injection Molding Method

#### 3.5.1. Tensile Properties

Mubarak et al. [137] investigated the mechanical properties of hybrid composites made from a combination of jute and man-made cellulose fibers with polypropylene, as summarized in Table 9. The manufacturing method used was pultrusion and injection molding, and the materials included a polypropylene block copolymer with ethylene, maleic acid anhydride, and 25% jute by weight. The results showed that the untreated composites had a tensile strength of 71.9 ± 0.4 MPa and Young’s modulus of 3.18 ± 0.05 GPa, while the treated composites had a tensile strength of 71 ± 0.6 MPa and Young’s modulus of 3.39 ± 0.06 GPa. The addition of jute and synthetic cellulose fibers to polypropylene has a positive effect on the mechanical properties of the resulting hybrid composites. The tensile strength and Young’s modulus of the composites increased compared to pure polypropylene. The hybrid composites of jute and synthetic cellulose fibers with polypropylene can be effectively produced using pultrusion and injection molding and have improved mechanical properties compared to pure polypropylene.

Zhili et al. [138] investigated the effect of different adhesive resin solutions on the tensile properties of composites fabricated from unidirectional carbon fiber and jute fiber. The jute fabric and carbon fiber fabric were cut into 360 mm × 160 mm and layered together. The jute fabric was placed in the outermost layer, and four layers of carbon fiber were used as the inner layer. The tensile strength was found within the range of 192.65 MPa to 225.89 MPa. The results indicated that the dosage of Polyamide203# and methyl silicone oil influenced the mechanical properties of the composites.

Rahman et al. [139] investigated the effect of post-treatment on the tensile properties of jute fiber-reinforced polypropylene composites. They used jute fibers, polypropylene and sodium periodate, and formic acid as materials and the injection molding method as the manufacturing method. The results showed that the tensile strength and Young’s modulus of the composites increased after pretreatment and post-treatment. The untreated composites had a tensile strength of 25.35 ± 0.45 MPa and Young’s modulus of 1.7 ± 0.05 GPa. After pretreatment, the tensile strength increased to 26.4 ± 1.1 MPa and Young’s modulus to 2 ± 0.05 GPa. The highest improvement in tensile properties was observed after pretreatment and post-treatment, with a tensile strength of 29.35 ± 0.65 MPa and Young’s modulus of 2.3 ± 0.05 GPa. The study demonstrated that post-treatment can significantly improve the tensile properties of jute fiber-reinforced polypropylene composites, making them more suitable for various applications.

Hong et al. [140] described the manufacture and testing of composite materials made from jute fibers and polypropylene. The jute fibers were treated with a silane solution to improve the bonding between the fibers and the polymer matrix. The composites were produced using injection molding. The results of the mechanical testing show that the treated composites have a slightly lower tensile strength and the same tensile modulus as the untreated composites. This indicates that the silane treatment did not have a significant effect on the overall mechanical properties of the composites. The tensile strength of the untreated composites was 39.85 ± 0.05 MPa, while the treated composites had a tensile strength of 35.60 ± 0.05 MPa. The tensile modulus was 8.24 ± 0.03 GPa for the untreated composites and 8.25 ± 0.04 GPa for the treated composites. The results suggest that while the silane treatment may improve the interfacial adhesion between the jute fibers and the polymer matrix, it does not have a significant impact on the overall mechanical properties of the composites. Further research is needed to determine the optimal conditions for salinization and to understand the full impact of the treatment on the mechanical properties of these composites.

#### 3.5.2. Flexural Properties

Mubarak et al. [137] also investigated the flexural/bending properties of the hybrid composites. The results showed that the untreated composites had a flexural strength of 68.5 ± 1.5 MPa and a flexural modulus of 2.72 ± 0.05 GPa, while the treated composites had a flexural strength of 71.5 ± 0.4 MPa and a flexural modulus of 2.77 ± 0.04 GPa, as summarized in Table 10. This indicates that the addition of jute and man-made cellulose fibers to polypropylene has a positive effect on the flexural properties of the composites. The treatment process also improved the flexural strength and modulus of the composites compared to the untreated composites. These findings suggest that the hybrid composites of jute and man-made cellulose fibers with polypropylene may have potential for applications requiring good flexural properties.

Zhili et al. [138] conducted the orthogonal test on jute-reinforced epoxy/resin matrix composites and showed a range of values for the flexural/bending properties. The study was based on three factors: A: content of T31 curing agent; B: content of polyamide203#; and C: content of methyl silicone oil. The results showed that the maximum flexural strength was 236.19 MPa, and the minimum flexural strength was 196.26 MPa. Similarly, the maximum flexural modulus was 1.992 GPa, and the minimum flexural modulus was 1.640 GPa. These results suggest that the combination of different levels of the three factors had an impact on the flexural properties of the jute-reinforced epoxy/resin matrix composites.

Rahman et al. [139] also evaluated the effect of post-treatment on the flexural properties of jute fiber-reinforced polypropylene composites. The results showed that both flexural strength and flexural modulus increased after pretreatment and post-treatment compared to untreated composites. The flexural strength of untreated composites was 25.35 ± 0.45 MPa, which increased to 26.4 ± 1.1 MPa after pretreatment and to 29.35 ± 0.65 MPa after pretreatment and post-treatment. Similarly, the flexural modulus increased from 1.7 ± 0.05 GPa for untreated composites to 2.0 ± 0.05 GPa after pretreatment and to 2.3 ± 0.05 GPa after pretreatment and post-treatment.

#### 3.5.3. Impact Properties

Mubarak et al. [137] found that the untreated composites had a Charpy impact strength of 79 ± 0.2 kJ/m^2^, while the treated composites had a Charpy impact strength of 72 ± 0.4 kJ/m^2^, as summarized in Table 10. This suggests that the addition of jute and man-made cellulose fibers to polypropylene has a positive effect on the impact strength of the composites, although the treatment process slightly decreased the impact strength compared to the untreated composites. Zhili et al. [138] also investigated the impact of different adhesive resin solutions on the impact strength of composites manufactured from unidirectional carbon and jute fiber. The maximum impact strength of the composites was found to be in the range of 1.6038 MPa to 1.9262 MPa

Rahman et al. [139] also studied the effect of post-treatment on the impact properties of jute fiber-reinforced polypropylene composites. The Charpy impact strength of jute fiber-reinforced polypropylene composites was found to have improved significantly through pretreatment and post-treatment. The untreated composite had a Charpy impact strength of 39.83 ± 0.17 J/m, while the pretreated composite had a strength of 41.75 ± 0.25 J/m.

#### 3.5.4. Thermal Properties

Mubarak et al. [137] also measured the heat distortion temperature (HDT). The results showed that the untreated composites had an HDT of 106 ± 0.3 °C, while the treated composites had an HDT of 112 ± 0.4 °C. This indicates that the addition of jute and man-made cellulose fibers to polypropylene had a positive effect on the thermal stability of the composites and that the treatment process further improved the HDT compared to the untreated composites.

#### 3.5.5. Hardness

Rahman et al. [139] also evaluated the effect of post-treatment on the hardness of jute fiber-reinforced polypropylene composites. The Rockwell hardness of the jute fiber-reinforced polypropylene composites was tested and found to increase with pretreatment and post-treatment. The untreated composite had a Rockwell hardness of 80, while the pretreated composite had a Rockwell hardness of 80.65 ± 0.35. The pretreated and post-treated composite showed the highest Rockwell hardness of 91 ± 0.25, which represents an improvement in hardness compared to the untreated and pretreated composites. These results indicate that pretreatment and post-treatment have a positive effect on the hardness of jute fiber-reinforced polypropylene composites.

#### 3.5.6. Water Absorption Properties

Rahman et al. [139] also investigated the effect of pretreatment and post-treatment on the water absorption properties of jute fiber-reinforced polypropylene composites. The results show that the water absorption increased from 0.68 ± 0.02% for the untreated composite to 0.76 ± 0.02% for the pretreated composite and further increased to 0.81 ± 0.02% for the pretreated and post-treated composite. These results indicate that both pretreatment and post-treatment have a significant impact on the water absorption properties of the composite, resulting in increased water absorption. Further studies are needed to understand the reasons behind this increase and to optimize the process parameters to achieve optimal water resistance in jute fiber-reinforced polypropylene composites.

### 3.6. Hot Press Method/Compression Molding Method

#### 3.6.1. Tensile Properties

Nabila et al. [141] fabricated jute fiber-reinforced composites using a hot press method that contained 40 wt.% of jute fibers. The jute fibers were treated with 5% NaOH to improve the bonding between the fibers and the polymer matrix. The results showed that the composites had a tensile strength of 38.2 ± 4.9 MPa and Young’s modulus of 3.2 ± 0.26 GPa, as summarized in Table 11. The tensile strength of the composites was found to be influenced by the weight fraction of jute fibers in the composite. These results suggest that there is an optimum level of jute fiber content in polypropylene composites for the highest tensile strength and Young’s modulus.

Subrata et al. [26] studied the mechanical properties of composites made of polypropylene and woven jute fabric. The composites were manufactured using a hot pressing molding method, and the jute fabric was present at a weight percentage of 50%. The results showed that the tensile strength of the composites was 67.23 ± 1.88 MPa, while Young’s modulus was 2.95 ± 0.55 GPa. These results indicate that the jute fiber reinforcement had a positive effect on the mechanical properties of the polypropylene composites. Arifuzzaman et al. [142] reported the results of a study on the tensile properties of composites made of woven jute fabric and poly(L-lactic acid) manufactured using a hot press molding method. The results showed that the tensile properties of the composites varied based on the type of jute reinforcement and the treatment applied to the jute fabric. The tensile strength of unidirectional jute was 55 ± 11.5 MPa, with Young’s modulus of 0.867 ± 0.02 GPa and a strain of 6.01%. For untreated woven jute in the wrap direction, the tensile strength was 81 ± 13.5 MPa, with Young’s modulus of 1.12 ± 0.034 GPa and a strain of 3.8%. The tensile properties of treated woven jute in the wrap direction showed a higher tensile strength of 87 ± 8.5 MPa, Young’s modulus of 1.42 ± 0.047 GPa, and a strain of 5.1%. For untreated woven jute in the weft direction, the tensile strength was 71 ± 8.7 MPa, with Young’s modulus of 0.78 ± 0.063 GPa and a strain of 4.1%. The treated woven jute in the weft direction had a tensile strength of 79.2 ± 9 MPa, Young’s modulus of 0.91 ± 0.057 GPa, and a strain of 4.2%. The results show that the tensile properties of the woven jute fabric reinforced poly (L-lactic acid) composites are influenced by the type of jute reinforcement and the treatment applied to the jute fabric. The treated woven jute in the wrap direction showed the best tensile properties, with the highest tensile strength and modulus. These results suggest that the treated woven jute in the wrap direction is a promising reinforcement for poly (L-lactic acid) composites for applications requiring high tensile strength and modulus.

Arobindo et al. [143] aimed to determine the mechanical properties of jute fiber-reinforced polypropylene laminate composite. The composite was manufactured by the hot press molding method using jute fibers with concentrations of 5% and 10% and lengths of 3 cm and 6 cm, along with polypropylene. The results showed that the tensile strength of the composite was affected by both the jute fiber concentrations and the length of the fibers. The highest tensile strength was observed in the composite with 10% jute fibers and a length of 3 cm, with a range of 10.23–17.86 MPa. The tensile strength of the composite with 5% jute fibers and a length of 6 cm was the lowest, with a range of 10.09-11.88 MPa. The results indicate that the use of higher jute fiber concentration and shorter fiber length can lead to a stronger composite. The results of this study suggest that the use of jute fiber reinforcement in polypropylene laminate composite can significantly improve the tensile strength of the composite. Shen et al. [144] showed that the tensile strength of jute fiber-reinforced polypropylene composites increases with the increase in fiber content up to 20%. The maximum tensile strength of 26.78 ± 0.64 MPa is achieved at 20% fiber content. Beyond this, the tensile strength starts to decrease, with a value of 27.42 ± 0.59 MPa at 35% fiber content and 24.96 ± 0.39 MPa at 50% fiber content. The results suggest that the addition of jute fiber to polypropylene enhances its tensile strength up to a certain limit. Beyond this limit, the tensile strength starts to decrease, which could be due to fiber clustering, fiber/matrix incompatibility, and reduced mobility of the matrix in the composite.

A jute fiber-reinforced polypropylene composite was manufactured by Keya et al. [145] using a hot pressing method. The results of the mechanical testing showed that the composite had a tensile strength of 45 MPa, a tensile modulus of 2.2 GPa, and an elongation at break of 11%. They found that the pineapple/pp composite had higher tensile strength than the jute/pp and okra/pp composites. Miah et al. [146] focused on the study of the mechanical and dielectric properties of composites made from low-density polyethylene (LDPE) and jute fabric. The composites were manufactured using heat press molding, and various concentrations of jute fiber were used (10%, 15%, 20%, 25%, and 30% wt.). The jute fibers were treated with a solution of 3% 2-hydroxyl ethyl methacrylate and 2% benzol peroxide in methanol. The results show that the tensile strength of the composites increased with increasing jute fiber concentration and the treated fibers resulted in higher tensile strengths compared to the untreated fibers. The maximum tensile strength of 25.12 MPa was observed at 25% wt. of treated jute fiber. Similarly, the elongation at break also increased with increasing jute fiber concentration, and the treated fibers resulted in higher elongation compared to the untreated fibers. The highest elongation of 50% was observed at 25 wt.% of treated jute fiber. Overall, the results suggest that the addition of jute fibers to LDPE significantly improves the mechanical properties of the composites. The treatment of the jute fibers with a solution of 2-hydroxyl ethyl methacrylate and benzol peroxide in methanol further enhances these properties.

Plateau [80] reinforced composites using the hot press method with chopped jute fiber in various lengths and weight fractions. Untreated or 20% NaOH-treated in polypropylene showed a positive effect of fiber weight percentage increment until a limit (10% wt.), and a reverse effect after that, and an increment in the length had a positive effect but a neutral effect by treatment in terms of tensile properties. Sudha and Thilagavathi [147] evaluated the effect of alkali treatment on the tensile properties of jute fabric-reinforced composites manufactured through handloom compression molding using vinyl ester resin. The results showed that the pull-out strength of the composites decreased after alkali treatment (5% NaOH), with a pull-out strength of 505 ± 165 MPa for untreated composites and 326 ± 150 MPa for treated composites.

Ranganathan et al. [148] investigated how long jute fiber-reinforced polypropylene composites made through compression molding responded to the addition of regenerated cellulose fibers as an impact modifier. Twisted jute yarn (30% in weight) and polypropylene made up the composites. Tension, flexure, and Izod impact tests were used to assess the composites’ mechanical characteristics. The composite had a tensile strength of 29.1 ± 1.1 MPa, Young’s modulus of 2.7 ± 0.103 GPa, and an elongation to break of 3.3 ± 1.0%, according to the data. Rashed et al. [149] examined the tensile strength of jute fiber-reinforced polypropylene composites in relation to process parameters. The composites were made using hot compression molding and jute fibers with 20% NaOH treatment. Testing of the mechanical characteristics involved tensile loading. The findings demonstrated that at lower fiber loadings (5% and 10%), untreated composites exhibited higher tensile strength than treated composites. Nevertheless, treated composites displayed marginally greater tensile strength at higher fiber loadings (15%). Moreover, the tensile strength of the composites was affected by their thickness, with thicker composites (4 mm) having lower tensile strength than thinner composites (1 mm and 2 mm).

Shajin et al. [150] investigated the impact of fiber length on the mechanical characteristics of jute fiber-reinforced polymer composites. Compression molding was used to create the composites from polyester and chopped jute fibers. Tensile strength declined with increasing fiber length, peaking at 29 MPa for 5 mm-long fibers and falling to 1.675 MPa for 25 mm-long fibers. The break load also decreased as fiber length increased. These findings imply that increasing the mechanical characteristics of jute fiber-reinforced composites is best accomplished by using shorter fiber lengths. Prasad et al. [151] examined the mechanical properties of polyester composites made by compression molding with banana and jute fiber reinforcement. Variable fiber volumes (5–25%) and fiber lengths were used to measure tensile strength (3mm and 5mm). The outcomes demonstrated that the tensile strength rose as the fiber volume increased. The composite with 25% fiber volume and 5mm fiber length had the maximum tensile strength, measuring 43.94 MPa. For all fiber volumes examined, an increase in fiber length was observed to increase tensile strength.

#### 3.6.2. Flexural and Impact Properties

The flexural and impact properties of JFRCs fabricated by the hot press method are shown in Table 12. Subrata et al. [26] also reported on the bending and impact properties of the JFRCs. The composites were made by varying the jute fiber content (40 to 55 wt.%), woven jute fabric, and polypropylene matrix and were manufactured using a hot press molding method. The results showed that there is optimum fiber content for which the composites showed better results. At 50 wt.% of fiber contents, the bending strength, modulus, and impact strength were 93.16 ± 5.92 MPa, 4.8 ± 0.31 GPa, and 15.59 kJ/m^2^. These results indicate that the addition of jute fiber to polypropylene has a positive effect on the bending properties of the composites up to a specific limit. Beyond this threshold, the mechanical properties deteriorated. The improvement in the bending strength and modulus suggests that the composites may have a higher resistance to deformation under bending loads, making them suitable for various applications that require high strength and stiffness.

Arifuzzaman et al. [142] evaluated the flexural and impact properties of woven jute fabric-reinforced poly(L-lactic acid) composites. The results showed that the flexural strength, modulus, and impact strength of the composites were influenced by both the type of jute reinforcement (unidirectional or woven) and the treatment applied to the jute fabric. For unidirectional jute, the flexural strength was found to be 67 ± 8.4 MPa, and the flexural modulus was 2.83 ± 1.1 GPa. The flexural strength and modulus of the untreated woven jute in the wrap direction were 82 ± 12.0 MPa and 4.3 ± 0.10 GPa, respectively. The treatment applied to the woven jute in the wrap direction improved the flexural strength and modulus, resulting in 121 ± 13.4 MPa and 5.3 ± 0.10 GPa, respectively. Similarly, the untreated woven jute in the weft direction had a flexural strength of 81 ± 9.4 MPa and a flexural modulus of 3.62 ± 0.08 GPa. After treatment, the flexural strength and modulus increased to 111 ± 8.1 MPa and 4.72 ± 0.05 GPa, respectively. When comparing the results of unidirectional jute and woven jute in both the untreated and treated forms, it was found that the treated woven jute fibers in the wrap direction had the highest flexural strength (121 ± 13.4 MPa) and flexural modulus (5.3 ± 0.10 GPa), while the untreated woven jute fibers in the weft direction had the lowest values. The results also showed that the unidirectional jute had an impact strength of 12.98 ± 1.1 kJ/m^2^, while the untreated woven jute in the wrap direction had an impact strength of 16.4 ± 1.8 kJ/m^2^. The treated woven jute in the wrap direction had a slightly higher impact strength of 18.1 ± 2.3 kJ/m^2^. The untreated woven jute in the weft direction had an impact strength of 14.3 ± 1.5 kJ/m^2^, while the treated woven jute in the weft direction had an impact strength of 16.6 ± 1.8 kJ/m^2^. Overall, the results suggest that the treatment applied to the woven jute can significantly improve the flexural and impact properties of the composites, making them more suitable for applications where high strength and stiffness are required.

Shen et al. [144] showed that the flexural strength of jute fiber-reinforced polypropylene composites increases with the increase in fiber content up to 20%. The maximum flexural strength of 35.46 ± 0.78 MPa is achieved at 20% fiber content. Beyond this, the flexural strength starts to decrease slightly, with a value of 36.40 ± 0.12 MPa at 35% fiber content and 35.02 ± 0.78 MPa at 50% fiber content. The results suggest that the addition of jute fiber to polypropylene enhances its flexural strength up to a certain limit. Beyond this limit, the flexural strength starts to decrease, which could be due to fiber clustering, fiber/matrix incompatibility, and reduced mobility of the matrix in the composite. Keya et al. [145] also showed that the flexural strength of the jute fiber-reinforced polypropylene composite is 54 MPa, and the flexural modulus is 4.1 GPa. They found that the okra/pp composites have much more bending strength than the jute/pp and pineapple/pp composites.

Miah et al. [146] also studied flexural properties, which show a similar trend as the tensile properties. The flexural strength of the composites increased with increasing jute fiber concentration and the treated fibers resulted in higher flexural strengths compared to the untreated fibers. The maximum flexural strength of 77.07 MPa was observed at 20 wt.% of treated jute fiber. However, it is noted that the flexural strength decreased at 30 wt.% of both treated and untreated jute fibers. This indicates that the addition of a high concentration of jute fibers may lead to reduced flexural strength. The results suggest that the addition of jute fibers to LDPE can significantly improve the flexural properties of the composites. The treatment of the jute fibers with a solution of 2-hydroxyl ethyl methacrylate and benzol peroxide in methanol further enhances these properties. However, it is important to consider the optimum concentration of jute fibers to balance the flexural strength and elongation at break.

Ranganathan et al. [148] found that the flexural strength, modulus, and impact strength were 47.1 7.1 MPa, 5.29 GPa, and 24.40 J/m for the long jute fiber-reinforced polypropylene composites made by compression molding. Jute fiber-reinforced polymer composites’ flexural strength was examined by Shajin et al. [150] at different fiber lengths ranging from 5 mm to 25 mm. The findings indicate that the flexural strength diminishes as fiber length increases. As the fiber length rose, the flexural strength values declined, with the lowest value of 21.5 MPa being recorded for the largest fiber length of 25 mm. The flexural strength values were found to be highest for the shortest fiber length of 5 mm, with a value of 64.66 MPa. The impact strength also found to be maximum for the shortest length of chopped jute fiber.

Prasad et al. [151] showed that with an increase in fiber volume fraction, the polyester composite reinforced with banana and jute fibers becomes more flexible. For the composite with a 20% fiber volume fraction, the maximum flexural strength was attained for both a 3 mm and a 5 mm thickness. Flexural strength for all thicknesses somewhat decreased at 25% fiber volume percentage. The composite with a 20% fiber volume fraction and 3 mm thickness had a maximum flexural strength of 55.89 MPa.

#### 3.6.3. Water Absorption Test

Subrata et al. [26] reported on the water absorption properties of woven jute fiber-reinforced polypropylene composites. The results showed that the water uptake of the composites in 24 h was 12.50 ± 0.50%. This result indicates the degree to which the composites absorbed water, which can have a significant effect on their properties and performance. A high water uptake rate can lead to dimensional instability, reduced strength, and a decrease in the overall durability of the composites.

#### 3.6.4. Thermal Properties

Nabila et al. [141] also reported on the thermal properties of jute fiber/polypropylene composites. The composites were produced using a hot press method and contained 40% jute fibers in weight. The jute fibers were treated with 5% NaOH to improve the bonding between the fibers and the polymer matrix. The results showed that the heat deflection temperature of the composites was 143.3 ± 1.14 °C. The heat deflection temperature is an important measure of a material’s resistance to deformation under a load at elevated temperatures. A higher heat deflection temperature indicates a material that can withstand higher temperatures before deformation occurs.

The results show that the jute fiber/polypropylene composites have a high heat deflection temperature, indicating that they have good thermal stability and may be useful in applications where elevated temperatures are encountered. Further research is needed to fully understand the thermal behavior of these composites and to determine their suitability for use in a range of high-temperature applications.

Sudha and Thilagavathi [147] investigated the effect of alkali treatment on the thermal conductivity of jute fabric-reinforced composites manufactured through handloom compression molding using vinyl ester resin. The results showed that the thermal conductivity of the treated composites increased significantly compared to the untreated composites, with a thermal conductivity of 106 ± 16 W/m.K for treated composites and 68 ± 17 W/m.K for untreated composites. This suggests that the alkali treatment can improve the thermal conductivity of jute fabric-reinforced composites.

#### 3.6.5. Compression Properties

Sudha and Thilagavathi [147] also evaluated the effect of alkali treatment on the compressive properties of jute fabric-reinforced composites manufactured through handloom compression molding using vinyl ester resin. The compressive strength increased twice after the alkali treatment.

## 4. Comparison of Mechanical Properties of JRFCs Based on Manufacturing Process

The choice of manufacturing process significantly influences the mechanical properties of JFRCs. Different fabrication processes affect void content, fiber/matrix adhesion, fiber orientation, and resin distribution. Among the various techniques, the hand lay-up process is most widely used because of its simplicity and cost-effectiveness. However, JFRCs fabricated using this method often suffer from inconsistencies in mechanical properties due to the presence of voids and non-uniform resin impregnation. Studies have shown that hand lay-up JFRCs exhibit moderate tensile strength and flexural properties. Their impact and compression strength tend to be lower due to the weak fiber/matrix adhesion caused by manual processing inconsistencies [25,112,123].

On the one hand, compression molding has been found to enhance the mechanical performance of JFRCs significantly. The application of high pressure during compression improves fiber wetting and reduces void formation, which leads to higher tensile and flexural strength compared to hand lay-up composites. Several studies have demonstrated that compression-molded JFRCs exhibit superior flexural modulus and impact resistance that is suitable for structural applications. Additionally, the improved interfacial bonding in compression-molded composites enhances their overall durability and resistance to mechanical loading [124,125].

On the other hand, injection molding is a highly efficient and scalable technique that offers the advantage of fabricating JFRCs with consistent mechanical properties due to controlled processing conditions. However, one of the primary challenges associated with injection-molded JFRCs is the fiber length reduction during processing, which can negatively impact tensile and flexural strength. Studies indicate that maintaining an optimal fiber length in injection-molded composites is crucial for achieving improved mechanical properties, as shorter fibers lead to a decrease in load-bearing capacity. Despite this limitation, injection molding provides superior impact resistance and dimensional accuracy compared to other techniques [57,152].

Resin transfer molding-fabricated JFRCs showed excellent mechanical properties due to their uniform fiber distribution and reduced void content. JFRCs manufactured through this process had enhanced tensile and flexural properties compared to hand lay-up and injection-molded composites. The controlled resin infusion process results in better fiber/matrix adhesion, which enhances the overall mechanical performance. Research findings suggest that surface treatment of jute fibers, combined with RTM processing, further improves tensile strength and modulus by ensuring better bonding at the fiber/matrix interface [153,154]. However, RTM is relatively expensive and requires precise control of injection parameters to achieve optimal results. The pultrusion process has also shown promising results in improving the tensile and flexural properties of JFRCs. The controlled pulling and curing process ensures uniform fiber alignment and resin impregnation, resulting in high stiffness and strength. Studies have demonstrated that pultruded JFRCs showed higher tensile modulus and impact strength compared to hand lay-up and compression-molded composites. However, the limitations of pultrusion include its applicability only to long, continuous profiles and the need for specialized equipment [90,155].

## 5. Recommendations and Future Research Directions

JFRCs have garnered significant attention as an environmentally friendly alternative to conventional synthetic composites in many industries. However, barriers remain in optimizing their physical and thermo-mechanical properties and suitability across various sectors. In the following sections, some recommendations are made for future research to enhance the properties of jute fiber composites and their recyclability.

### 5.1. Fiber Treatment and Surface Alteration Techniques

An essential determinant of the mechanical characteristics of JFRCs is the interfacial adhesion between the jute fibers and the polymer matrix. Numerous studies [119,156,157,158,159,160] have demonstrated that surface treatments, including alkali treatment, silane coupling agents, and other chemical modifications, can greatly augment the adhesion between fibers and the matrix. This, in turn, results in enhanced mechanical characteristics, namely in terms of tensile and flexural strength. However, previous research indicates discrete information on the fiber treatment methods. For instance, some researchers found that fiber treatment using a certain amount of aqueous solution of NaOH results in the maximum mechanical strength, but others showed a different percentage for the maximum mechanical strength. There are various methods of treating jute fiber for optimum properties of the resulting composites. Subsequent investigations should prioritize the optimization of these treatment procedures to achieve a harmonious equilibrium between enhancing the integration of fibers with the matrix and preserving the intrinsic characteristics of jute fibers. Advanced surface modification methods, including plasma treatment [161] and enzymatic treatment [162], also offer opportunities to enhance JFRC performance. Plasma treatment increases functional groups on the fiber surface, boosting reactivity with the matrix, while enzymatic treatment removes non-cellulosic components, improving compatibility without compromising structural integrity. Investigating the synergistic effects of these treatments could lead to composites with superior mechanical characteristics. Since jute fabrics are available in various forms, such as unidirectional fiber, yarn, roving, and mats with various weaving patterns, comprehensive research is needed for the optimization of treatment methods for various types of jute fiber and fabric.

### 5.2. Fiber Hybridization

Hybrid composites, comprising jute fibers with other natural or synthetic fibers, have demonstrated promise in addressing some constraints exhibited by single-fiber composites [163,164,165,166]. Synthesizing jute fiber with glass or carbon fibers can greatly enhance the mechanical properties of the composites. Moreover, hybridization enables the customization of composite characteristics to fulfill certain application criteria for industrial applications. Further studies are needed to evaluate the advancement of hybrid composites that not only boost mechanical properties but also preserve the ecological advantages associated with the use of natural fibers. This may involve using bio-derived synthetic fibers or incorporating alternative natural fibers like hemp or flax. Additionally, studies should focus on optimizing fiber content and combinations to maximize the performance of the hybrid composites. For laminates, numerical research may be conducted to find an application-oriented hybrid composite with the incorporation of jute fiber-reinforced composite laminas, which would lead to reduced use of synthetic fiber in composite manufacturing.

### 5.3. Manufacturing Process Optimization

The manufacturing process has great importance in determining the final characteristics of jute-based composites. Each fabrication process (hand lay-up, compression molding, injection molding, resin transfer molding, etc.) has its own pros and cons. Compression molding is renowned for its ability to manufacture composites with excellent mechanical characteristics. However, it needs meticulous regulation of production parameters to minimize flaws such as porosity, agglomeration, and uneven distribution of fibers. Although injection molding is suitable for mass manufacturing, it frequently encounters difficulties associated with the length and orientation of fibers, which can affect the mechanical properties of the composites. Direct fiber-feeding injection molding shows promise in improving fiber dispersion and matrix saturation [167]. Future research should focus on optimizing these processes to reduce defects and enhance fiber distribution within the matrix. Additionally, improvements in autonomous production methods, such as robotic lay-up systems and automated curing techniques, can improve the quality of JFRCs while reducing the labor-intensive characteristics of traditional manufacturing processes, therefore improving the scalability and cost-effectiveness of JFRC production. The manufacturing methods of JFRCs are complicated and require a significant amount of time and labor, which leads to difficulties in manufacturing customized products. Therefore, new manufacturing methods should be developed to reduce the manufacturing difficulties associated with the manufacturing of jute fiber-reinforced polymer composites to make these composites popular in various industries.

### 5.4. Utilization of Bio-Based Resins

JFRCs can be made more environmentally friendly by substituting bio-based resins with traditional petroleum-based resins. Bio-based resins, such as polylactic acid (PLA) and bio-polyethylene, are organic compounds obtained from renewable sources that have comparable or even better mechanical characteristics than conventional resins [168,169,170,171]. Extensive research is required to determine the compatibility of jute fibers and bio-based resins to guarantee robust interfacial bonding and achieve the best possible composite performance. Future research should prioritize the development and characterization of novel bio-based resin systems tailored for integration with natural fibers such as jute. This entails examining the extended-term resilience, heat resistance, and capacity to break down naturally of these composites in different environmental circumstances. The incorporation of bio-based resins with sophisticated fiber treatments has the potential to facilitate the creation of completely sustainable composite materials with improved performance properties. It should be noted that petroleum-based resins are not easily biodegradable, but bio-based resins have good biodegradability. Owing to the nature of bio-based resins, more research is needed to explore the full potential of jute fiber-reinforced composites.

### 5.5. Exploration of Nanotechnology in JFRCs

The application of nanotechnology presents promising opportunities for augmenting the characteristics of JFRCs. The integration of nanomaterials, including nano clays, carbon nanotubes, and graphene, into the polymer matrix or as external layers on jute fibers can greatly enhance the mechanical, thermal, and barrier characteristics of the composites [172,173,174,175]. Furthermore, these improvements have the potential to broaden the scope of uses for JFRCs, especially in high-performance domains like the aerospace and automotive industries. Currently, research is being conducted on the utilization of nanomaterials, but more research is needed to fully understand the interactions between nanoparticles and jute fibers, together with their influence on the general characteristics of the composites. Furthermore, it is also necessary to investigate the feasibility of integrating nanomaterials into JFRCs and the possible ecological and health consequences of employing nanomaterials in composite production.

### 5.6. Assessment of Life Cycle, Carbon Footprint, Recyclability, and Environmental Impact

Although the environmental advantages of employing natural fibers such as jute are well-established, it is necessary to conduct thorough life cycle evaluations of JFRCs to measure their cumulative environmental effect in comparison to conventional composites because JFRCs often consist of petroleum-based resins for manufacturing. These investigations should include the complete lifespan of the composite, starting from the extraction and processing of raw materials to the disposal or recycling at the end of its useful life. The carbon footprint should also be assessed for future net zero emission targets set by the United Nations. The aim of future research may be to establish a life cycle assessment strategy for natural fiber composites, considering energy use, greenhouse gas emissions, water usage, waste generation, etc. Additionally, the recycling method for jute fiber-reinforced composites is lacking in the literature, and the possibility of recycling JFRCs after their lifespan, including the practicality of repurposing jute fibers and the retrieval of the polymer matrix, can be investigated.

### 5.7. Development of Smart JFRCs

The incorporation of smart technologies into JFRCs may be a recent field for further investigation. By integrating sensors, actuators, or other functional materials, smart composites can provide supplementary capabilities, including self-healing, damage detection, and environmental monitoring [176,177]. For instance, the integration of piezoelectric materials into JFRCs has the potential to facilitate the advancement of composites that produce electrical energy when subjected to mechanical stress. Studies may include the identification of appropriate smart materials that exhibit compatibility with jute fibers and can be smoothly incorporated into the composite matrix system. Additionally, the progress of smart JFRCs will heavily depend on the development of manufacturing methods that enable the incorporation of these materials without compromising the mechanical characteristics of the composites.

### 5.8. Customization for Particular Applications

JFRCs can be customized for particular uses by modifying the fiber content, orientation, and matrix composition. Further investigation is needed to evaluate the advancement of application-specific JFRCs, with an emphasis on enhancing the composite characteristics for certain sectors, including automotive, construction, and packaging. This may need the application of sophisticated modeling and simulation methods to forecast the performance of JFRCs under different loading conditions and environmental variables. Additionally, partnerships among academia, industry, and government agencies can play an important role in promoting the commercialization of tailored JFRCs. This has the potential to expedite the establishment of industry standards and certification procedures for natural fiber composites.

## 6. Concluding Remarks

These studies present a comprehensive analysis of the fabrication process of JFRCs and their effects on the mechanical properties. Based on the thorough literature review, several concluding remarks can be summarized as follows.

- The mechanical properties of JFRCs are greatly influenced by fiber treatment, matrix type, fiber orientation, and the particular production technique employed. Experimental research shows that alkali treatment enhances jute fibers’ affinity for the polymer matrix, which improves the mechanical properties of the composites. However, careful regulation of alkali treatment is essential to prevent fiber degradation.
- Manufacturing processes have a significant role in determining the quality and performance of JFRCs. Each process, including hand lay-up, compression molding, injection molding, and RTM, has unique benefits and constraints. Although the hand lay-up process is economical and adaptable, it is labor-intensive and susceptible to variations that may lead to flaws, such as voids and inadequate fiber or matrix distribution.
- Compression molding and RTM show better fiber dispersion and resin impregnation. Nevertheless, these processes need meticulous regulation of processing parameters, and any deviations might result in substantial material defects. Improvements in automated and scalable production methods are essential for improving the industrial feasibility of JFRCs.
- Although JFRCs have vast potential, they encounter several obstacles that must be resolved to enable their broader implementation in industrial applications. The major obstacles are the absorption of moisture, the quality of fibers, and the significant duration of the production process cycle. Moisture absorption is a major concern, as it can result in the degradation of the composite material over time. These obstacles can be mitigated by using proper treatment or hybridization with other fibers or particles.
- In order to save production time without sacrificing the mechanical and thermal properties of the composites, future studies should focus on the optimization of the process parameters, development of new manufacturing methods, life cycle assessment, recyclability, techno-economic analysis, etc.

## Figures and Tables

**Table 1:** Tensile properties (hand lay-up method).

Ref.	Jute Fiber/Fabric Type	Resin	Treatment	Percentage of Fiber in Composites		Tensile Strength, MPa	Tensile Modulus, GPa	Tensile Elongation
[25]	Fiber length 5–6 mm	Polyester	5% NaOH	18% wt.		9.24	0.811	1.14 mm
			10% NaOH			7.92	-	-
		Epoxy	5% NaOH			12.46	1.064	1.17 mm
			10% NaOH			10.5	-	-
[112]	Bi-directional jute fiber mat	Epoxy	Untreated	12% wt.		71.67	0.96	-
				24% wt.		88.87	3.03	-
				36% wt.		97.99	3.81	-
				48% wt.		110	4.45	-
[113]	Woven fabric	Epoxy	Untreated	25% wt.		26.53	6.32	-
[114]	Main and cross-directional woven textile	Epoxy	Heat treatment			189.479		
[115]	Continuous jute fibers (5 layers)	Polyester	Dry	50% wt.		34.87	1.989	-
			Wet—water			35.23	2.23	-
[116]	Raw jute fiber reed	Un-saturated polyester	30% jute batching oil in water emulsion	25% wt.		80 ± 13.39	3.68 ± 0.48	4.5 ± 0.55%
				35% wt.		106 ± 16.30	4.83 ± 0.63	5.2 ± 0.83%
				44% wt.		122 ± 31.11	5.56 ± 0.67	4.8 ± 0.54%
	Jute silver			25% wt.		71 ± 11.93	3.24 ± 0.65	4.8 ± 0.59%
				35% wt.		89 ± 9.74	4.46 ± 0.45	5.4 ± 0.48%
				44% wt.		109 ± 16	4.89 ± 0.55	4.7 ± 0.54%
[117]	Long jute fibers	Epoxy	Untreated	70% wt.		419	-	-
[118]	Plain weave jute fabrics	Polypropylene	0.4% NaOH	30% wt.		16.73	-	-
				40% wt.		22.31	-	-
				45% wt.		24.21	-	-
				50% wt.		21.55	-	-
				60% wt.		20.15	-	-
				65% wt.		18.50	-	-
[119]	Unidirectional jute fiber	Epoxy	Untreated	8% wt.		45.28 ± 0.45	3.18 ± 0.40	-
				10% wt.		54.35 ± 4.88	5.66 ± 0.65	-
				12% wt.		78.38 ± 1.01	9.9 ± 1.61	-
			Chemically treated	8% wt.		51.25 ± 4.88	11.9 ± 0.55	-
				10% wt.		67.97 ± 2.99	12.38 ± 1.38	-
				12% wt.		84.46 ± 4.99	13 ± 0.67	-
[120]	Bi-axial woven jute fabric	Epoxy	Untreated	44.3 ± 2.2% vol.		52.1	5.184	1.6%
[121]	Woven jute fiber plies	Polyester	Untreated	20% vol.	Uni	42.236	3.973	0.011 m/m
					Multi	21.685	-	0.529 m/m
[122]	Cross-plied	Epoxy	Untreated	-		16.62	0.664	-
[123]	Combed unidirectional	Epoxy	Untreated	40% vol.		179	-	-
			5% NaOH			432	-	-
[124]	Matted jute fabric	Epoxy	Untreated	-		46.7	-	-
			20% NaOH			97.5	-	-
[125]	Bi-directional woven jute	Polyester	Untreated	30% vol.		30	2.1	-
				33% vol.		35	2.5	-
				37% vol.		46	3	-
				40% vol.		60	4	-
[126]	Twine form fabric weaved in bi-directional mat	Epoxy	Untreated	30% vol.		39.75 ± 0.97	39.75 ± 0.97	-
			Alkalized			39.08 ± 3.35	3.60 ± 0.24	-
			Alkalized + Silanized			43.07 ± 3.80	3.77 ± 0.23	-
[127]	Jute fabric	Epoxy	Untreated	30% vol.		45.628	-	-
			Alkalized			50.19	-	-
[128]	Short jute fibers	Polyester	Untreated	16% wt.		30.6 ± 2.30	3.368 ± 0.18	1.209 ± 0.08%
			5% NaOH			34.2 ± 2.91	3.946 ± 0.22	1.221 ± 0.09%
			PLA-coated			31.6 ± 2.83	3.489 ± 0.19	1.212 ± 0.07%
			Alkalized + PLA-coated			36.6 ± 3.12	3.991 ± 0.23	1.324 ± 0.08%
[129]	Matted jute	Epoxy	5% wt. NaOH	10% wt.		28.33 ± 1.05	0.6246 ± 0.0325	-
				20% wt.		31.71 ± 2.11	0.8486 ± 0.0516	-
				30% wt.		33.04 ± 0.46	1.0453 ± 0.0379	-
				40% wt.		33.72 ± 1.73	1.2284 ± 0.0846	-
				50% wt.		34.26 ± 2.59	1.1785 ± 0.1085	-
[130]	Long jute fiber	Epoxy	Untreated	1 layer		11.02	0.90	-
				2 layers		42.73	1.06	-
				3 layers		53.69	1.40	-
[131]	Non-woven + fabric + non-woven fibers with Unsaturated polyester, soy flour resin	no Alkyd Resin	Untreated	-		2.83	-	1.57%
		5% Alkyd Resin				24.87	-	3.46%
		10% Alkyd Resin				25.79	-	5.27%

**Table 2:** Compression properties (hand lay-up method).

Ref.	Jute Fiber/Fabric Type	Resin	Treatment	Percentage of Fiber in Composite	Compressive Strength, MPa	Compressive Modulus, GPa	Strain, %
[120]	Bi-axial woven	Epoxy	Untreated	44.3 ± 2.2% vol.	40.2	3.523	-
[132]	Woven jute fabric	Un-saturated polyester	Untreated	25% wt.	56.09	0.75	4.72
			4% NaOH		57.42	0.44	12.97
			5% NaOH		69.01	0.88	7.77
			7% NaOH		55.63	0.65	8.59
[125]	Bi-directional woven jute	Polyester	Untreated	30% vol.	58	-	-
				33% vol.	54	-	-
				37% vol.	49	-	-
				40% vol.	40	-	-

**Table 3:** Flexural properties (hand lay-up method).

Ref.	Fiber Type	Resin	Treatment	Percentage of Fiber	Flexural Strength, MPa	Flexural Modulus, GPa	Flexural Elongation
[25]	Fiber length 5–6 mm	Polyester	5% NaOH	18% wt.	44.71	1.91	5.5 mm
			10% NaOH		40.5	-	-
		Epoxy	5% NaOH		39.08	3.08	2.1 mm
			10% NaOH		32.5	-	-
[112]	Bi-directional jute fiber mat	Epoxy	Untreated	12% wt.	28.61	0.59	-
				24% wt.	34.79	0.73	-
				36% wt.	51.22	1.24	-
				48% wt.	55.8	3.02	-
[113]	Woven fabric	Epoxy	Untreated	25% wt.	66.67	5.78	-
[114]	Main and cross-directional woven textile	Epoxy	Heat treatment		208.705	-	-
[115]	Continuous jute fibers (5 layers)	Polyester	Dry	50% wt.	67.56	2.59	-
			Wet by water		68.89	3.121	-
[116]	Raw jute fiber reed	Un-saturated polyester	30% jute batching oil in water emulsion	25% wt.	102 ± 16.23	9.42 ± 1.31	2.27 ± 0.15%
				35% wt.	124 ± 17.97	11.6 ± 1.65	3.49 ± 0.28%
				44% wt.	145 ± 21.94	15.41 ± 2.22	3.18 ± 0.31%
	Jute silver			25% wt.	85 ± 20.16	7.56 ± 1.36	2.61 ± 0.60%
				35% wt.	103 ± 14.64	10.64 ± 1.41	2.66 ± 0.57%
				44% wt.	112 ± 17.30	13.24 ± 2.12	2.57 ± 0.48%
[118]	Plain weave jute fabrics	Polypropylene	0.4% NaOH	30% wt.	34.75	-	-
				40% wt.	42.49	-	-
				45% wt.	44.26	-	-
				50% wt.	39.31	-	-
				60% wt.	38.05	-	-
				65% wt.	36.14	-	-
[132]	Woven jute fabric	Un-saturated polyester	Untreated	25% wt.	39.63	1.56	2.52%
			4% NaOH		47.91	1.77	2.70%
			5% NaOH		57.16	1.49	3.81%
			7% NaOH		56.75	2.13	2.66%
[122]	Cross-plied	Epoxy	Untreated	-	57.22	8.956	
[123]	Combed unidirectional	Epoxy	Untreated	40% vol.	85	-	-
			5% NaOH		89	-	-
[124]	Matted jute fabric	Epoxy	Untreated	-	62.4	-	-
			20% NaOH		80.1	-	-
[126]	Twine form fabric weaved in bi-directional mat	Epoxy	Untreated	30% vol.	64.30 ± 5.50	4.63 ± 0.42	-
			Alkalized		56.31 ± 5.68	3.53 ± 0.34	-
			Alkalized + Silanized		50.62 ± 2.31	3.53 ± 0.34	-
[127]	Jute fabric	Epoxy	Untreated	30% vol.	81.12	-	-
			Alkalized		90.89	-	-
[128]	Short jute fibers	Polyester	Untreated	16% wt.	58.17 ± 3.14	3.931 ± 0.17	1.861 ± 0.11%
			5% NaOH		78.27 ± 4.12	5.872 ± 0.25	2.414 ± 0.12%
			PLA-coated		67.68 ± 3.26	4.420 ± 0.19	2.090 ± 0.12%
			Alkalized + PLA-coated		79.76 ± 4.67	6.231 ± 0.31	2.512 ± 0.14%
[129]	Matted jute	Epoxy	5% wt. NaOH	10% wt.	44.2 ± 2.65	0.7363 ± 0.0458	-
				20% wt.	49.6 ± 4.32	1.0248 ± 0.0276	-
				30% wt.	68.8 ± 4.49	1.2906 ± 0.0241	-
				40% wt.	81.8 ± 6.78	1.2583 ± 0.0546	-
				50% wt.	97.8 ± 5.25	1.0133 ± 0.179	-
[130]	Long jute fiber	Epoxy	Untreated	1 layer	31.3	1.42	-
				2 layers	56.32	2.03	-
				3 layers	76.52	3.02	-
[131]	Non-woven + fabric + non-woven fibers with Unsaturated polyester, soy flour resin	no Alkyd Resin	Untreated	-	2.83	-	-
		5% Alkyd Resin			24.87	-	-
		10% Alkyd Resin			25.79	-	-

**Table 4:** Impact strength (hand lay-up method).

Ref.	Fiber Type	Resin	Treatment	Percentage of Fiber in Composites	Type of Impact Test	Impact Strength
[25]	Fiber length 5–6 mm	Polyester	5% NaOH	18% wt.	Charpy	3.25 J
			10% NaOH			2.75 J
		Epoxy	5% NaOH			2.63 J
			10% NaOH			2 J
[112]	Bi-directional jute fiber mat	Epoxy	Untreated	12% wt.	-	3.048 J
				24% wt.		3.929 J
				36% wt.		4.528 J
				48% wt.		4.875 J
[113]	Woven fabric	Epoxy	Untreated	25% wt.		80 J/m^2^
[115]	Continuous Jute fibers (5 layers)	Polyester	Dry	50% wt.	Charpy	6.14 J
			Wet by water			7.23 J
[117]	Long jute fibers	Epoxy	Untreated	70% wt.	Charpy	3 J
					Izod	2 J
[118]	Plain weave jute fabrics	Polypropylene	0.4% NaOH	30% wt.	Charpy	54.42 kJ/m^2^
				40% wt.		61.27 kJ/m^2^
				45% wt.		61.78 kJ/m^2^
				50% wt.		51.62 kJ/m^2^
				60% wt.		49.09 kJ/m^2^
				65% wt.		44.39 kJ/m^2^
[122]	Cross-plied	Epoxy	Untreated	-	Izod	13.44 kJ/m^2^
[123]	Combed unidirectional	Epoxy	Untreated	40% vol.	Charpy	480.76 kJ/m^2^
			5% NaOH			76.92 kJ/m^2^
[126]	Twine form fabric weaved in bi-directional mat	Epoxy	Untreated	30% vol.	-	143.06 ± 22.39 J/m
			Alkalized			171.68 ± 18.28 J/m
[127]	Jute fabric	Epoxy	Untreated	30% vol.	Izod	69.5 J/cm^2^
			Alkalized			88.5 J/cm^2^
[128]	Short jute fibers	Polyester	Untreated	16% wt.	Izod	5.10 ± 0.32 kJ/m^2^
			5% NaOH			2.29 ± 0.16 kJ/m^2^
			PLA-coated			3.95 ± 0.21 kJ/m^2^
			Alkalized + PLA-coated			5.30 ± 0.29 kJ/m^2^
[129]	Matted jute	Epoxy	5% wt. NaOH	10% wt.	-	20.35 ± 0.2 kJ/m^2^
				20% wt.		20.98 ± 0.24 kJ/m^2^
				30% wt.		21.84 ± 0.05 kJ/m^2^
				40% wt.		22.65 ± 1.1 kJ/m^2^
				50% wt.		23.87 ± 1.36 kJ/m^2^

**Table 5:** Rockwell hardness (hand lay-up method).

Ref.	Fiber Type	Resin	Treatment	Percentage of Fiber in Composites	Rockwell Hardness Value
[25]	Fiber length 5–6 mm	Polyester	5% NaOH	18% wt.	44
			10% NaOH		41.67
		Epoxy	5% NaOH		42
			10% NaOH		41
[112]	Bi-directional jute fiber mat	Epoxy	Untreated	12% wt.	70.68
				24% wt.	74.01
				36% wt.	78.54
				48% wt.	85.5
[117]	Long jute fibers	Epoxy	Untreated	70% wt.	40
[131]	Non-woven + fabric + non-woven fibers with unsaturated polyester, soy flour resin	no Alkyd Resin	Untreated	-	44.6
		5% Alkyd Resin			63
		10% Alkyd Resin			66.60

**Table 6:** Tensile and flexural properties (vacuum molding).

Ref.	Jute Fiber/Fabric Type	Resin	Treatment	Percentage of Fiber in Composites	Tensile Strength, MPa	Tensile Modulus, GPa	Flexural Strength, MPa	Flexural Modulus, GPa
[133]	Unidirectional	Epoxy	-	52% wt.	216 ± 1.02	31 ± 1.34	158 ± 18.90	18 ± 1.92
[134]	Bi-directional woven	Vinylester	Untreated	-	-	-	103 ± 6	6.6 ± 0.5
			5% NaOH		-	-	83 ± 6	5.5 ± 0.2

**Table 7:** Flexural and impact properties (heating in hollow cylindrical glass).

Ref.	Fiber Type	Resin	Treatment	Percentage of Fiber in Composites	Flexural Strength, MPa	Flexural Modulus, GPa	Charpy Impact Strength
[135]	White jute fibers	Vinyl-ester	Untreated	35% vol.	199.10 ± 7.6	11.89 ± 0.62	22.10 ± 2.79 kJ/m^2^
			5% NaOH for 4 h		238.90 ± 17.60	14.69 ± 0.85	21.92 ± 3.84 kJ/m^2^
			5% NaOH for 8 h		204.20 ± 1.20	12.32 ± 0.35	19.97 ± 0.78 kJ/m^2^

**Table 8:** Tensile and impact properties (extrusion process).

Ref.	Jute Fiber/Fabric Type	Resin	Treatment	Percentage of Fiber in Composites	Tensile Strength, MPa	Tensile Modulus, GPa	Izod Impact Strength
[136]	Uniaxial jute yarn	Polypropylene	-	6% vol.	28.11	1.32	25.77 kJ/m^2^
				12% vol.	29.24	1.61	28.40 kJ/m^2^
				18% vol.	27.31	1.87	29.33 kJ/m^2^
				23% vol.	27.98	2.04	17.89 kJ/m^2^
				29% vol.	33.12	2.03	17.47 kJ/m^2^
				34% vol.	33.56	2.18	13.19 kJ/m^2^
				45% vol.	34.46	2.28	11.46 kJ/m^2^

**Table 9:** Tensile properties (Injection molding).

Ref.	Jute Fiber/Fabric Type	Resin	Treatment	Percentage of Fiber in Composites	Tensile Strength, MPa	Tensile Modulus, GPa	Tensile Elongation
[137]	Yarned jute	Polypropylene	Untreated	25% wt.	71.9 ± 0.4	3.18 ± 0.05	-
			Maleic Acid Anhydride		71 ± 0.6	3.39 ± 0.06	-
[138]	Jute fiber	Epoxy, Polyamide Resin,	Untreated	-	192.65 -225.89	-	-
[139]	Chopped jute fibers	Polypropylene	Untreated	20% wt.	25.726	1.682	-
				25% wt.	25.359	1.71	-
				30% wt.	24.18	2.137	-
				35% wt.	23.536	2.221	-
			Oxidized	20% wt.	27.092	1.714	-
				25% wt.	26.374	1.999	-
				30% wt.	25.158	2.234	-
				35% wt.	24.33	2.313	-
			Oxidized and post-treated	20% wt.	29.473	1.864	-
				25% wt.	29.365	2.03	-
				30% wt.	28.998	2.29	-
				35% wt.	27.201	2.398	-
[140]	Short jute fiber	Polypropylene	Untreated	1% wt.	26.778	0.807	5.429 mm
				5% wt.	-	1.189	-
				10% wt.	-	1.766	-
				15% wt.	35.856	2.153	2.102 mm
			Silanized	1% wt.	29.162	0.844	5.811 mm
				5% wt.	-	1.158	-
				10% wt.	-	1.819	-
				15% wt.	37.049	2.193	1.957 mm

**Table 10:** Flexural and impact properties (injection molding).

Ref.	Fiber Type	Resin	Treatment	Percentage of Fiber in Composites	Flexural Strength, MPa	Flexural Modulus, GPa	Charpy Impact Strength
[137]	Yarned Jute	Polypropylene	Untreated	25% wt.	68.5 ± 1.5	2.72 ± 0.05	79 ± 0.2 kJ/m^2^
			Maleic Acid Anhydride		71.5 ± 0.4	2.77 ± 0.04	47.2 ± 4.2 kJ/m^2^
[138]	Jute fiber	Epoxy, Polyamide Resin,	Untreated	-	196.26–236.19	1.640–1.992	1.60–1.9 MPa
[139]	Chopped jute fibers	Polypropylene	Untreated	20% wt.	45.499	1.928	18.513 MPa
				25% wt.	47.145	2.136	31.199 MPa
				30% wt.	47.191	2.246	31.245 MPa
				35% wt.	45.515	2.404	19.307 MPa
			Oxidized	20% wt.	49.459	2.406	23.426 MPa
				25% wt.	49.665	2.487	34.435 MPa
				30% wt.	49.689	2.521	34.480 MPa
				35% wt.	47.175	2.553	23.261 MPa
			Oxidized and post-treated	20% wt.	53.979	2.532	39.243 MPa
				25% wt.	54.385	2.766	48.694 MPa
				30% wt.	54.350	2.995	48.379 MPa
				35% wt.	45.499	3.101	31.11 MPa

**Table 11:** Tensile properties (hot press method/compression molding).

Ref.	Jute Fiber/Fabric Type	Resin	Treatment	Percentage of Fiber in Composites	Tensile Strength, MPa	Tensile Modulus, GPa	Tensile Elongation
[141]	Matted jute	Polypropylene	5% NaOH	30% in weight	33.5	2.8	3.09%
				40% in weight	38.2	3.2	2.95%
				50% in weight	36.38	3.17	2.84%
[26]	Woven jute fabric	Polypropylene	Untreated	40% in weight	53.12	2.51	-
				45% in weight	58.40	2.79	-
				50% in weight	68.27	2.94	-
				55% in weight	56.29	2.77	-
[142]	Unidirectional	Poly L-lactic acid	-	-	55 ± 11.5	0.867 ± 0.02	6.01%
	Woven jute fabric—Wrap		Untreated	52 yarns per 100 mm	81 ± 13.5	1.12 ± 0.034	3.8%
			Treated		87 ± 8.5	1.42 ± 0.047	5.1%
	Woven jute fabric—Weft		Untreated	44 yarns per 100 mm	71 ± 8.7	0.78 ± 0.063	4.1%
			Treated		79.2 ± 9	0.91 ± 0.057	4.2%
[143]	Chopped—3 cm, 1-Ply	Polypropylene	Untreated	5%	10.44 ± 0.62	-	-
	Chopped—3 cm, 2-Ply				12.01 ± 1.66	-	-
	Chopped—3 cm, 4-Ply				11.54 ± 2.86	-	-
	Chopped—6 cm, 1-Ply				10.09 ± 1.34	-	-
	Chopped—6 cm, 2-Ply				11.88 ± 2.51	-	-
	Chopped—6 cm, 4-Ply				10.16 ± 2.8	-	-
	Chopped—9 cm, 1-Ply				9.68 ± 2.48	-	-
	Chopped—9 cm, 2-Ply				11.36 ± 2.73	-	-
	Chopped—9 cm, 4-Ply				10.34 ± 3.40	-	-
	Chopped—3 cm, 1-Ply			10%	10.23 ± 1.61	-	-
	Chopped—3 cm, 2-Ply				17.86 ± 0.62	-	-
	Chopped—3 cm, 4-Ply				12.47 ± 3.05	-	-
	Chopped—6 cm, 1-Ply				10.21 ± 1.61	-	-
	Chopped—6 cm, 2-Ply				13.48 ± 1.48	-	-
	Chopped—6 cm, 4-Ply				12.16 ± 2.30	-	-
	Chopped—9 cm, 1-Ply				9.92 ± 2.22	-	-
	Chopped—9 cm, 2-Ply				13.65 ± 2.16	-	-
	Chopped—9 cm, 4-Ply				11.93 ± 4.12	-	-
[144]	Chopped jute fiber	Polypropylene	NaOH	5% wt.	23.08 ± 0.94	-	-
				20% wt.	26.78 ± 0.64	-	-
				35% wt.	27.42 ± 0.59	-	-
				50% wt.	24.96 ± 0.3	-	-
[145]	Bleached jute fabric	Polypropylene	Untreated	45% wt.	45	2.2	11%
[146]	Jute fabric	Low-density polyethylene	Untreated	10% wt.	15.05	-	30.51%
				15% wt.	17.23	-	33.02%
				20% wt.	19.05	-	35.38%
				25% wt.	20.05	-	36.06%
				30% wt.	19.09	-	26.02%
			3% 2-hydroxyl ethyl methacrylate and 2% benzol peroxide treated	10% wt.	17.11	-	32.17%
				15% wt.	21.26	-	35.23%
				20% wt.	23.68	-	41.32%
				25% wt.	25.12	-	50 %
				30% wt.	23.43	-	48.10%
[80]	Chopped jute fibers—1 mm long	Polypropylene	Untreated	5% wt.	23.5	-	-
				10% wt.	26		
				15% wt.	20.4		
			Treated 20% NaOH	5% wt.	19		
				10% wt.	25		
				15% wt.	24		
	Chopped jute fibers—2 mm long		Untreated	5% wt.	33	-	-
				10% wt.	26		
				15% wt.	26.4		
			20% NaOH	5% wt.	30		
				10% wt.	32.4		
				15% wt.	26		
	Chopped jute fibers—4 mm long		Untreated	5% wt.	26	-	-
				10% wt.	25.04		
				15% wt.	25.04		
			20% NaOH	5% wt.	33		
				10% wt.	28		
				15% wt.	27		
[147]	Plain woven jute fibers	Vinyl ester resin	Untreated		4.5 ± 0.2	-	-
			5% NaOH		= 8.3 ± 0.6	-	-
[148]	Twisted jute yarn	Polypropylene	Untreated	30% in wt.	29.1 ± 1.1	2.7 ± 0.103	3.3 ± 1.0%
[149]	Chopped jute fiber—1 mm	Polypropylene	Untreated	5% wt.	23.29 ± 0.34	-	-
				10% wt.	26.39 ± 0.34	-	-
				15% wt.	22.13 ± 0.34	-	-
			20% NaOH	5% wt.	18.99 ± 0.11	-	-
				10% wt.	25.24 ± 0.11	-	-
				15% wt.	24.42 ± 0.11	-	-
	Chopped jute fiber—2 mm		Untreated	5% wt.	26.55 ± 0.34	-	-
				10% wt.	31.71 ± 0.34	-	-
				15% wt.	27.05 ± 0.34	-	-
			20% NaOH	5% wt.	30.52 ± 0.11	-	-
				10% wt.	33.15 ± 0.11	-	-
				15% wt.	26.58 ± 0.11	-	-
	Chopped jute fiber—4 mm		Untreated	5% wt.	25.13 ± 0.34	-	-
				10% wt.	26.11 ± 0.34	-	-
				15% wt.	25.13 ± 0.34	-	-
			20% NaOH	5% wt.	32.77 ± 0.11	-	-
				10% wt.	28.05 ± 0.11	-	-
				15% wt.	27.41 ± 0.11	-	-
[150]	Chopped jute fiber—5 mm	Polyester	Untreated	30% wt.	29	-	-
	Chopped jute fiber—10 mm				9.46	-	-
	Chopped jute fiber—15 mm				8.68	-	-
	Chopped jute fiber—20 mm				2.08	-	-
	Chopped jute fiber—25 mm				1.675	-	-
[151]	Chopped jute fiber—3 mm	Polyester	-	5% wt	17.09	-	-
				10% wt.	22.49	-	-
				15% wt.	23.45	-	-
				20% wt.	35.15	-	-
				25% wt	43.94	-	-
	Chopped jute fiber—5 mm			5% wt	10.68	-	-
				10% wt.	14.93	-	-
				15% wt.	16.59	-	-
				20% wt.	24.88	-	-
				25% wt.	39.28	-	-

**Table 12:** Flexural and impact properties (hot press method/compression molding).

Ref.	Fiber Type	Resin	Treatment	Percentage of Fiber in Composites	Flexural Strength, MPa	Flexural Modulus, GPa	Impact Strength
[26]	Woven jute fabric	Polypropylene	Untreated	40% in weight	74.83	-	8.99 kJ/m^2^
				45% in weight	78.77	-	13.07 kJ/m^2^
				50% in weight	94.43	-	14.59 kJ/m^2^
				55% in weight	77.32	-	11.92 kJ/m^2^
[142]	Unidirectional	Poly L-lactic acid	-	-	67 ± 8.4	2.83 ± 1.1	12.98 kJ/m^2^
	Woven jute fabric—Wrap		Untreated	52 yarns per 100 mm	82 ± 12.0	4.3 ± 0.10	16.4 kJ/m^2^
			Treated		121 ± 13.4	5.3 ± 0.10	18.1 kJ/m^2^
	Woven jute fabric— Weft		Untreated	44 yarns per 100 mm	81 ± 9.4	3.62 ± 0.08	14.3 kJ/m^2^
			Treated		111 ± 8.1	4.72 ± 0.05	16.6 kJ/m^2^
[144]	Chopped jute fiber	Polypropylene	NaOH	5% wt.	31.16 ± 1.89	-	-
				20% wt.	35.46 ± 0.78	-	-
				35% wt.	36.40 ± 0.12	-	-
				50% wt.	35.02 ± 0.78	-	-
[145]	Bleached jute fabric	Polypropylene	Untreated	45% wt.	54	4.1	0.61 kJ/m^2^
[146]	Jute fabric	low-density polyethylene	Untreated	10% wt.	22.23	-	-
				15% wt.	39.81	-	-
				20% wt.	48.62	-	-
				25% wt.	48.01	-	-
				30% wt.	47.14	-	-
			3% 2-hydroxyl ethyl Methacrylate and 2% benzol peroxide-treated	10% wt.	27.07	-	-
				15% wt.	61.14	-	-
				20% wt.	77.07	-	-
				25% wt.	73.05	-	-
				30% wt.	20.15	-	-
[148]	Twisted jute yarn	Polypropylene	Untreated	30% in wt.	47.1 ± 7.1	= 5.269 ± 0.482	24.4 ± 3.1 J/m
[150]	Chopped jute fiber—5 mm	Polyester	Untreated	30% wt.	64.66	-	0.61 J
	Chopped jute fiber—10 mm				30.46	-	0.51 J
	Chopped jute fiber—15 mm				25.56	-	0.48 J
	Chopped jute fiber—20 mm				22.86	-	0.45 J
	Chopped jute fiber—25 mm				21.5	-	0.39 J
[151]	Chopped jute fiber—3 mm	Polyester	-	5% wt.	15.75	-	-
				10% wt.	26.16		
				15% wt.	42.51		
				20% wt.	55.89		
				25% wt.	53.65		
	Chopped jute fiber—5 mm		-	5% wt.	12.12	-	-
				10% wt.	21.90		
				15% wt.	24.87		
				20% wt.	41.23		
				25% wt.	38.15		

## Data Availability

No new data were created or analyzed in this study.
